# *Desulfitobacterium* contributes to the microbial transformation of 2,4,5‐T by methanogenic enrichment cultures from a Vietnamese active landfill

**DOI:** 10.1111/1751-7915.13301

**Published:** 2018-08-16

**Authors:** Ute Lechner, Dominique Türkowsky, Thi Thu Hang Dinh, Hassan Al‐Fathi, Stefan Schwoch, Stefan Franke, Michelle‐Sophie Gerlach, Mandy Koch, Martin von Bergen, Nico Jehmlich, Thi Cam Ha Dang

**Affiliations:** ^1^ Institute of Biology/Microbiology Martin‐Luther University Halle‐Wittenberg Halle Germany; ^2^ Department of Molecular Systems Biology Helmholtz Centre for Environmental Research – UFZ Leipzig Germany; ^3^ Vietnamese Academy of Science and Technology Institute of Biotechnology Hanoi Vietnam; ^4^ Institute of Chemistry/Food and Environmental Chemistry Martin‐Luther University Halle‐Wittenberg Halle Germany; ^5^ Present address: Vietnamese Academy of Science and Technology Graduate University of Science and Technology Hanoi Vietnam

## Abstract

The herbicide 2,4,5‐trichlorophenoxyacetic acid (2,4,5‐T) was a major component of Agent Orange, which was used as a defoliant in the Vietnam War. Little is known about its degradation under anoxic conditions. Established enrichment cultures using soil from an Agent Orange bioremediation plant in southern Vietnam with pyruvate as potential electron donor and carbon source were shown to degrade 2,4,5‐T via ether cleavage to 2,4,5‐trichlorophenol (2,4,5‐TCP), which was further dechlorinated to 3,4‐dichlorophenol. Pyruvate was initially fermented to hydrogen, acetate and propionate. Hydrogen was then used as the direct electron donor for ether cleavage of 2,4,5‐T and subsequent dechlorination of 2,4,5‐TCP. 16S rRNA gene amplicon sequencing indicated the presence of bacteria and archaea mainly belonging to the *Firmicutes*,* Bacteroidetes*,* Spirochaetes, Chloroflexi* and *Euryarchaeota*. *Desulfitobacterium hafniense* was identified as the dechlorinating bacterium. Metaproteomics of the enrichment culture indicated higher protein abundances of 60 protein groups in the presence of 2,4,5‐T. A reductive dehalogenase related to RdhA3 of *D. hafniense* showed the highest fold change, supporting its function in reductive dehalogenation of 2,4,5‐TCP. Despite an ether‐cleaving enzyme not being detected, the inhibition of ether cleavage but not of dechlorination, by 2‐bromoethane sulphonate, suggested that the two reactions are catalysed by different organisms.

## Introduction

During the Vietnam War (1961–1971), about 100 million litres of defoliating herbicides was sprayed by the US Army in order to remove forest canopies and destroy crops in Central and South Vietnam. The most notorious herbicide used was Agent Orange, consisting of a 1:1 mixture of the butyl esters of 2,4,5‐trichlorophenoxyacetic acid (2,4,5‐T) and 2,4‐dichlorophenoxyacetic acid (2,4‐D). During chemical synthesis, 2,4,5‐T was also contaminated by varying levels of 2,3,7,8‐tetrachlorodibenzo‐*p*‐dioxin (Stellman *et al*., 2003). The Bien Hoa airbase was the major staging point for the Operation ‘Ranch Hand’ in South Vietnam and served as storage site for the barrels containing the herbicides. Large spills during handling led to a high herbicide and dioxin contamination of soils and sediments in the vicinity of Bien Hoa (Dwernychuk *et al*., 2002; Mai *et al*., 2007). An active landfill (3384 m^3^ of herbicide‐ and dioxin‐contaminated soil subdivided into the four bioremediation cells BH1‐BH4) was established and operated for 40 months (Dang and Nguyen, 2012). The treatment strategy aimed to develop anoxic conditions to allow the establishment of polychlorinated dibenzo‐*p*‐dioxins‐dechlorinating communities (see the Description of the bioactive landfill in Bien Hoa in the Supporting Information). During this treatment, the average total concentration of polychlorinated dibenzo‐*p*‐dioxins was initially 10 000 ng toxicity equivalents (TEQ) (kg dry weight)^−1^ and was decreased to 52 ng TEQ (kg dry weight)^−1^ within 27 months as indicated by the analysis of 16 composite samples taken from all four cells (Dang, 2013). As inferred from the results of a pilot plant in Da Nang, Central Vietnam operating for 6 months, where approximately 4 g (kg dry weight)^−1^ of the Agent Orange herbicides rapidly declined under aerobic and anaerobic conditions (Allen, 2015), an ongoing degradation of the phenoxyacetate herbicides during the treatment process in Bien Hoa was also expected.

Aerobic biodegradation of the phenoxyacetic acid herbicides 2,4‐D and 2,4,5‐T has been well characterized on the molecular and biochemical levels (e.g. Daubaras *et al*., 1996; Schlömann, 2002; Pérez‐Pantoja *et al*., 2008; Xu *et al*., 2013). Degradation starts with an oxygenase‐catalysed cleavage of the ether bond (e.g. Danganan *et al*., 1995; Müller *et al*., 2006) followed by ring hydroxylation and cleavage as part of the modified *ortho*‐ or hydroquinone pathway, for the degradation of chlorinated aromatic compounds (Reineke, 2001). A metaproteomics approach identified key enzymes of this pathway in soil columns percolated with 2,4‐D, supporting its ubiquitous presence in soil communities (Benndorf *et al*., 2007).

Biodegradation of 2,4‐D and 2,4,5‐T under anoxic conditions has not been extensively studied. The reductive dechlorination of 2,4,5‐T has been reported for sewage sludge, pond sediment, a methanogenic aquifer and soils (Mikesell and Boyd, 1984; Suflita *et al*., 1984; De Weerd *et al*., 1986; Gibson and Suflita, 1986, 1990; Bryant, 1992; Chang *et al*., 1998). The first 2,4,5‐T dechlorination products were identified as 2,4‐D and 2,5‐D, but monochlorophenoxyacetic acids, tri‐, di‐ and monochlorophenols, as well as phenol, were also observed. Some samples were reported wherein cleavage of the ether bond preceded dechlorination of the chlorophenols formed (Eder, 1980; Gibson and Suflita, 1993). The microbial composition of these anaerobic, 2,4‐D‐ or 2,4,5‐T‐converting enrichment cultures has not been reported, and no pure anaerobic culture that grows at the expense of 2,4,5‐T conversion is available.

In this study, we investigated the anaerobic transformation of 2,4,5‐T by enrichment cultures obtained from the active landfill in Vietnam, with the aim of identifying the degradation pathways and the organisms involved. The influence of electron donors and the inhibitor 2‐bromoethane sulphonate (BrES) on the two reactions, the cleavage of the ether bond and the dechlorination of the intermediate 2,4,5‐TCP were studied. Our analysis of the microbial community involved identified candidates responsible for catalysing the individual reactions.

## Results

### Transformation of 2,4,5‐T to 2,4,5‐TCP and 3,4‐dichlorophenol by the enrichment cultures

**Figure 1:**
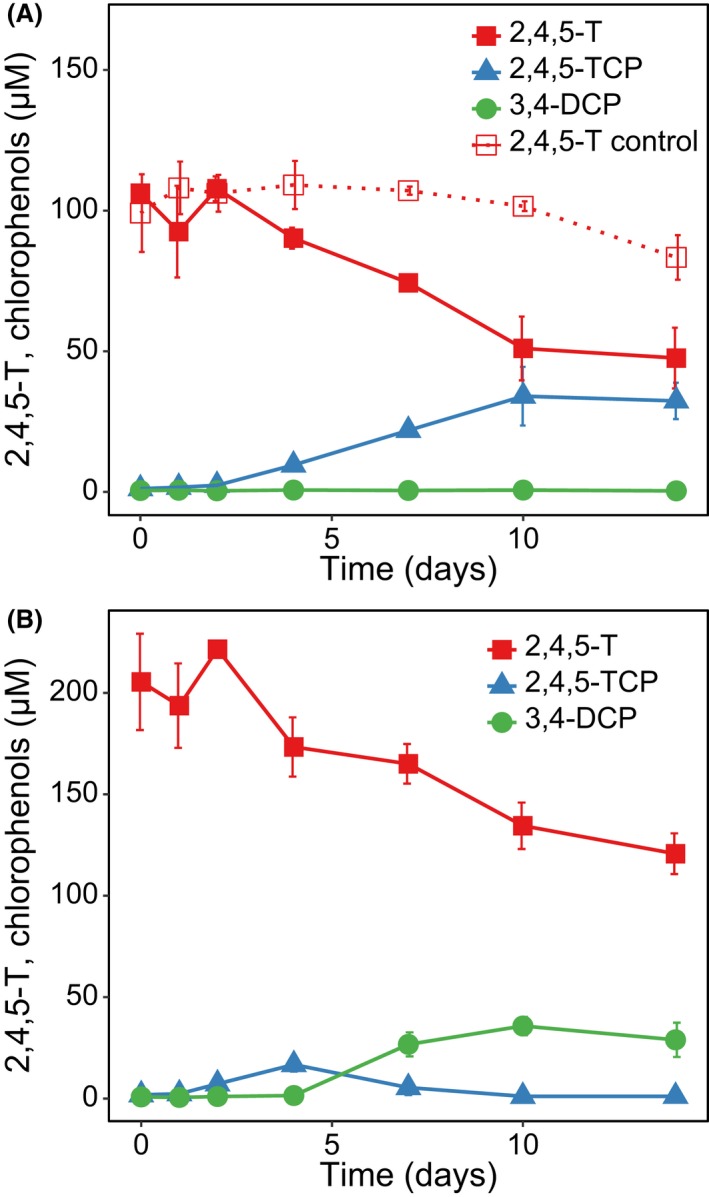
Degradation of 2,4,5‐T by enrichment culture BH1.1 supplemented with 2.5 mM lactate (A) or pyruvate (B) as determined by HPLC (2,4,5‐T) and GC (chlorophenols) measurement. The dashed line indicates an abiotic control. Note the different initial concentrations of 2,4,5‐T in both sets of triplicate cultures. The main products were 2,4,5‐TCP and 3,4‐DCP.

The primary microcosms and the first subculture obtained from sample sites BH1.1, BH 4.3 and BH4.4 were shown to degrade 2,4,5‐T rapidly to different chlorinated phenols in the presence of pyruvate and lactate (5 mM each) (Dinh *et al*., 2015) (see also the Description of the primary and secondary enrichment cultures in Supporting Information). These cultures were repeatedly transferred. Table S1 summarizes the enrichment procedure including minor changes in the medium composition. Starting from the fifth transfer, the amount of electron donors was reduced (i.e. subcultures received either lactate or pyruvate, each at 2.5 mM) with the intention of reducing the number of fermenting bacteria and proportionately enriching for bacteria that might benefit from the bioconversion of 2,4,5‐T. In the 6^th^ subculture, the products of 2,4,5‐T transformation were recorded by HPLC and GC measurements. In general, after a lag time of 1–2 days the initial concentration of 2,4,5‐T (100–200 μM) decreased by 40–90 μM over a period of 10–14 days and subsequently levelled off (representatively shown for enrichment culture BH1.1 supplemented with lactate or pyruvate in Fig. 1A and B). The cultures supplemented with lactate (BH1.1 and BH4.4) formed 2,4,5‐TCP as the main product in almost stoichiometric proportions, whereas those supplemented with pyruvate (BH1.1, BH4.3 and BH4.4) showed a transient formation of 2,4,5‐TCP and the accumulation of 3,4‐dichlorophenol (3,4‐DCP). Samples from selected time points were additionally analysed using one replicate of each culture variant by GC‐MS to confirm and quantify the identified products and to analyse whether further degradation products were formed (Table S2). In the lactate‐supplemented cultures of BH1.1 and BH4.4, 2,4,5‐TCP was the main product that accumulated over time (40–60 μM). Minor products (<0.5 μM) in these cultures were 2,5‐DCP and 3,4‐DCP. This result confirmed that only minor 2,4,5‐TCP dechlorination activities existed in the lactate‐fed cultures. In contrast, in the pyruvate‐supplemented cultures, the presence of both 2,4,5‐TCP and 3,4‐DCP was confirmed. In addition, 1–2 μM 4‐chlorophenol and about 0.5–1 μM phenol were detected in some cultures. No, or only trace concentrations (<0.24 μM) of the dichlorinated phenoxyacetic acids 2,4‐D or 2,3‐/3,4‐D or of the monochlorinated 2‐ or 3‐chlorophenoxyacetic acid were determined in some cultures (Table S2). This strongly suggests that 2,4,5‐T itself was not significantly targeted by dechlorination reactions in these long‐term enrichment cultures.

In all further experiments, the nearly stoichiometric formation of 2,4,5‐TCP and 3,4‐DCP from 2,4,5‐T was confirmed and was used to monitor the degradation process while disregarding the traces of other minor products. To test whether 2,4,5‐TCP was the direct precursor of 3,4‐DCP, a subculture of BH4.4/pyruvate was supplemented with either 100 μM 2,4,5‐T or 30 μM 2,4,5‐TCP (2,4,5‐TCP is relatively toxic and could not be supplied at concentrations ≥ 40 μM; see also Madsen and Aamand, 1992). In both experiments, 3,4‐DCP was formed. 2,4,5‐TCP was dechlorinated to 3,4‐DCP within 2 weeks, whereas formation of 3,4‐DCP from 2,4,5‐T was significantly lower, indicating that the cleavage of the ether bond is the rate‐limiting step of the overall process, restricting the availability of 2,4,5‐TCP for the subsequent reductive dechlorination (Fig. S1).

### Desulfitobacterium is involved in 2,4,5‐TCP dechlorination and pyruvate fermentation

**Figure 2:**
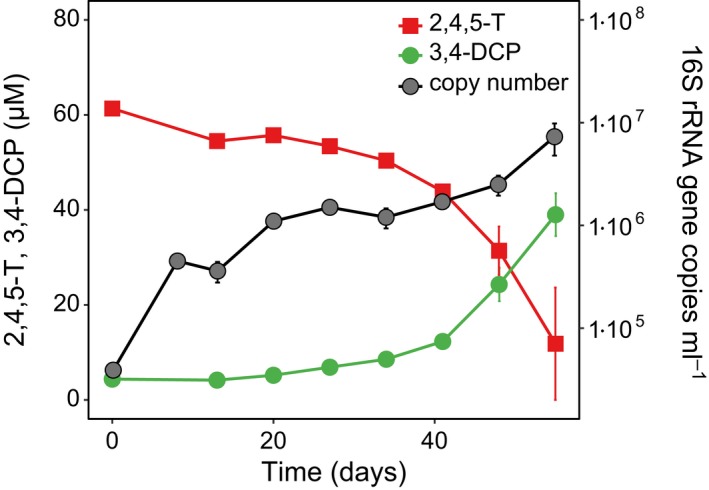
Increase in the copy number of *Desulfitobacterium* sp. 16S rRNA genes during 2,4,5‐T conversion to 3,4‐DCP in culture BH1.1. The concentration of 2,4,5‐TCP did not exceed 2 μM over the course of the experiment (not shown). After 8 days, pyruvate and citrate were completely converted to acetate (data not shown). Mean values and SD from duplicate cultures and triplicate qPCR analyses are given.

**Figure 3:**
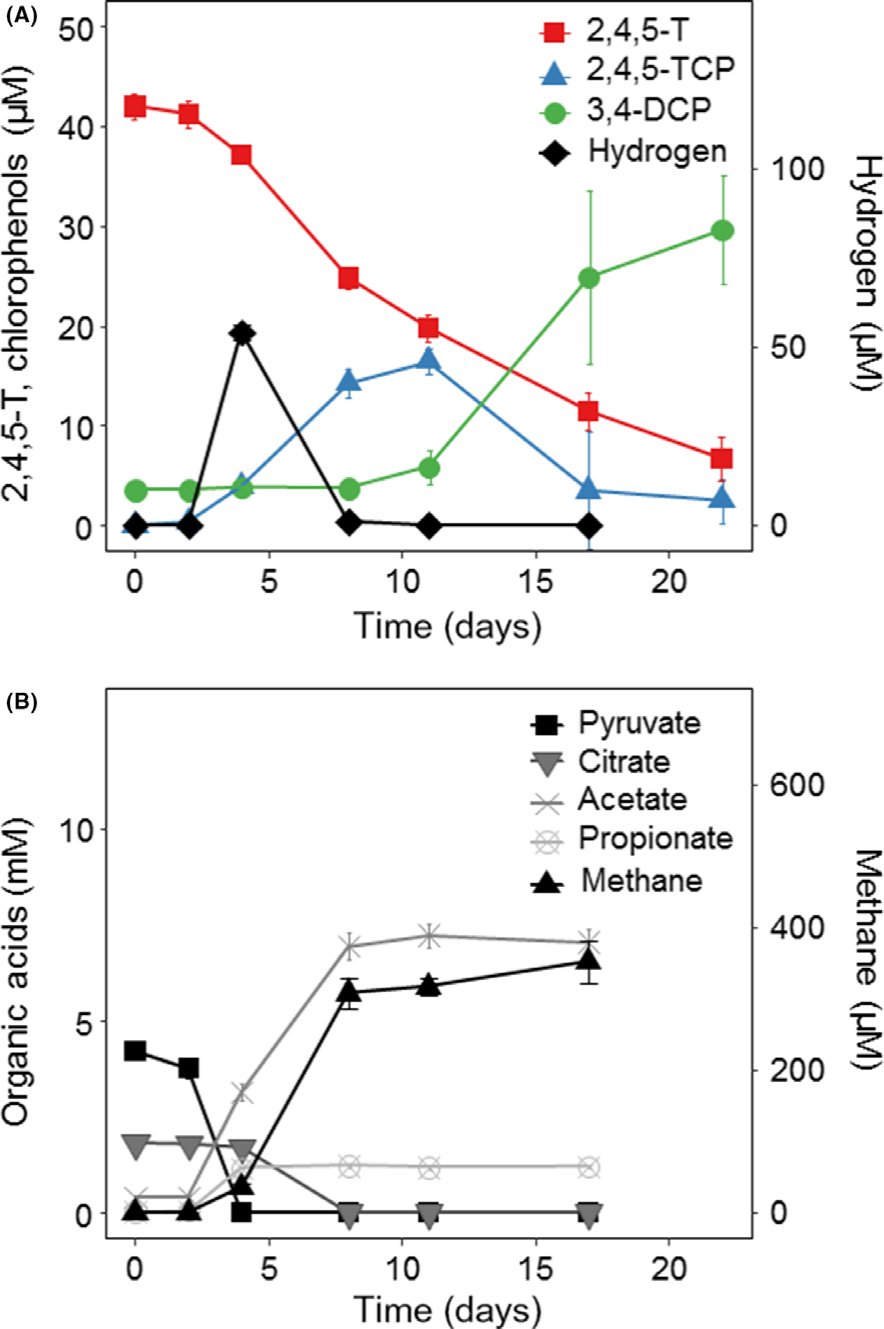
Transformation of 2,4,5‐T (A) and fermentation of pyruvate and citrate (B) in enrichment culture BH1.1 cultivated in the absence of yeast extract. Intermediate formation of hydrogen is shown for clarity in A, whereas formation of other fermentation products and methane is given in B. Mean value and SD of triplicate cultures.

**Figure 4:**
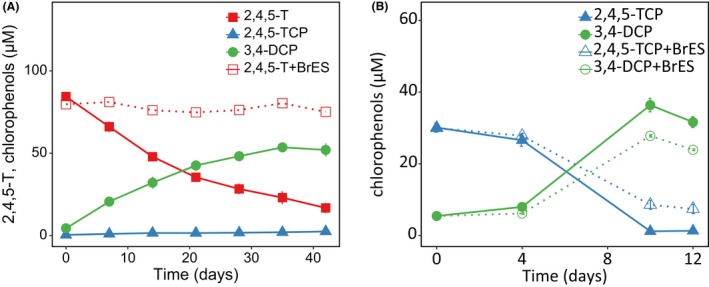
Influence of 5 mM 2‐bromoethane sulphonate (BrES) on ether cleavage of 2,4,5‐T and dechlorination of 2,4,5‐TCP. BH1.1 cultures were incubated with 2,4,5‐T (A) or 2,4,5‐TCP (B). Results are shown for triplicate cultures (mean value and SD). Data from cultures with BrES are indicated by dashed lines.

All enrichment cultures except for BH1.1/lactate were further transferred. The observed incomplete conversion of 2,4,5‐T was overcome in these next transfers by increasing the amount of pyruvate or lactate to 5 mM (e.g. Figs 2, 3 and 4). Using genus‐specific primers, duplicate cultures of all enrichment cultures were tested for the presence of well‐known organohalide‐respiring bacteria. With primers targeting the 16S rRNA genes of the obligate organohalide‐respiring *Dehalococcoides* and *Dehalobacter spp*., no PCR products were obtained from any culture when compared to reference strains as positive control. However, *Desulfitobacterium* sp. was detected in the three enrichment cultures supplemented with pyruvate (BH1.1, BH4.3, and BH4.4), which transformed 2,4,5‐T to 3,4‐DCP. No PCR products were obtained from the non‐dechlorinating enrichment culture with lactate (BH4.4, Fig. S2). The involvement of *Desulfitobacterium* in the dechlorination of 2,4,5‐TCP to 3,4‐DCP was confirmed by qPCR targeting *Desulfitobacterium* 16S rRNA genes in a pyruvate subculture (BH1.1, Fig. 2). The copy number of *Desulfitobacterium* 16S rRNA genes increased by more than one order of magnitude at the beginning of cultivation (days 0–8), long before the onset of 2,4,5‐T transformation to 3,4‐DCP. This finding and the observation that the initially added 5 mM pyruvate was completely consumed by 8 days (data not shown) suggest that initial growth occurred by pyruvate fermentation. During the phase of the highest rate of 3,4‐DCP formation (days 34–55), the copy number of *Desulfitobacterium* 16S rRNA genes further increased by an order of magnitude, which suggests growth by organohalide respiration. The enrichment culture BH1.1/pyruvate showed the highest long‐term stability and fastest 2,4,5‐T conversion to 3,4‐DCP via 2,4,5‐TCP. This culture was used for all subsequent studies.

### Chlorinated phenoxyacetic acids are transformed faster than the non‐chlorinated phenoxyacetic acid

To test the spectrum of chlorinated phenoxyacetic acids transformed, the enrichment culture BH1.1 was incubated with 5 mM pyruvate and five selected phenoxyacetic acids. Ether cleavage started for all compounds within 4 days except for the non‐chlorinated phenoxyacetic acid. After 11 days, 2,4,5‐T was completely converted to 3,4‐DCP, while 2,4‐D was converted via 2,4‐DCP to 4‐chlorophenol. The ether bond of 3,4‐dichloro‐ and 2‐chlorophenoxyacetic acid was also cleaved leading to 3,4‐DCP and 2‐chlorophenol, respectively, neither of which was further dechlorinated. The non‐chlorinated phenoxyacetic acid was cleaved only slowly, resulting in the formation of phenol (Table 1).

**Table 1:** Conversion of the non‐chlorinated and different chlorinated phenoxyacetic acids after 11 days within enrichment culture BH1.1

Phenoxyacetic acid, initial concentration (μM)^a^	Product(s) (mol‐% of the initial phenoxyacetic acid)^a^
2,4,5‐T (77.7 ± 1.2)	2,4,5‐TCP (traces), 3,4‐DCP (88.1 ± 3.1)
2,4‐D (89.6 ± 3.4)	4‐chlorophenol (73.2 ± 3.1)
3,4‐dichlorophenoxyacetic acid (57.2 ± 2)	3,4‐DCP (106.1 ± 2.3)
2‐chlorophenoxyacetic acid (56.6 ± 2.7)	2‐chlorophenol (95.4 ± 4.5)
Phenoxyacetic acid (77.8 ± 4.8)	phenol (19.4 ± 1.7)

**a**. Mean value and SD for triplicate cultures, grown in the presence of 5 mM pyruvate and without yeast extract.

### Fermentation of pyruvate preceded 2,4,5‐T transformation

Acetate (2.5 mM) as potential carbon source had been routinely added to the enrichment culture from the beginning. Because pyruvate‐fed cultures also converted 75 μM 2,4,5‐T in the absence of added acetate (e.g. Figs 2 and 3), the latter was subsequently omitted from the medium. The enrichment cultures were also supplemented with yeast extract to meet the needs of potentially unknown growth requirements. The conversion of 2,4,5‐T and the fermentation of pyruvate (4.2 mM) were now monitored in cultures with and without yeast extract addition (Fig. 3A and B, Fig. S3A and B). In both culture variants, pyruvate was fermented to acetate, propionate and hydrogen within 4 days. Subsequently, the citrate, which was added as a component of the reducing agent titanium citrate (the stock solution contains titanium and citrate in a 1:2 ratio according to its preparation), was degraded. Methane formation started with the appearance of hydrogen (day 4) and accumulated to ~0.3 mM after 8 days, when the hydrogen concentration dropped below the detection limit. Interestingly, the onset of 2,4,5‐T degradation at day 4 coincided with the depletion of pyruvate and the appearance of hydrogen, whereas dechlorination of the formed 2,4,5‐TCP started after 8 and 11 days in the presence and absence of yeast extract respectively. The overall kinetics and mass balances of pyruvate and citrate fermentation and of 2,4,5‐T ether cleavage were almost the same with and without yeast extract. In contrast, 2,4,5‐TCP accumulated to a slightly higher concentration and was more slowly dechlorinated to 3,4‐DCP in the absence of yeast extract (Fig. 3, Fig. S3), suggesting a positive influence of yeast extract on the dechlorinating bacterium. The mass balances of pyruvate and citrate fermentation and of methanogenesis were calculated (Fig. S4).

### Microbial community structure based on 16S rRNA gene sequencing

In order to obtain an overview of the community composition, DNA was extracted from three independent BH1.1 cultures with different histories of yeast extract addition. One set of cultures received yeast extract continuously over 11 transfers (+/+YE). Another set of cultures did not receive yeast extract in the last two transfers. These served as inoculum for two sets of cultures with (−/+YE) and without (−/−YE) yeast extract. DNA was extracted in the phase of active dechlorination, and the 16S rRNA gene amplicons were sequenced. The genus level distribution of 16S rRNA genes based on the respective read numbers is shown in the Fig. S5. The most abundant genera belonged to the *Firmicutes*,* Bacteriodetes*,* Spirochaetes*,* Chloroflexi* and *Euryarchaeota* (Fig. S5A). A broader diversity of genera, however, mainly of the same phyla, was represented by lower read frequencies (Fig. S5B), and among these, *Desulfitobacterium* was the only organohalide‐respiring bacterium that has been described to date. Most genera, including *Desulfitobacterium*, positively responded to the continuous presence of yeast extract (+/+YE) or to its re‐addition (−/+YE), which might explain the observed more robust 2,4,5‐TCP dechlorination in the presence of yeast extract. Omission of yeast extract (−/−YE) led to a distinct increase in *Pelosinus*,* Geosporobacter* and *Anaerotruncus*, three members of the *Firmicutes*. Considerable numbers of reads representing methanogenic species were detected. *Methanosarcina* strongly decreased in the absence of yeast extract (<11 reads under −/− and −/+YE conditions, Fig. S4A), whereas *Methanobacterium* was still represented by 160–340 reads after one or two transfers without yeast extract (−/+YE and −/−YE) (Fig. S5B), supporting the fact that it was not strictly dependent on yeast extract addition.

### 2‐Bromoethane sulphonate inhibits ether cleavage of 2,4,5‐T

To test the role of methanogens in the degradation process, BrES was added to cultures supplemented with pyruvate, yeast extract and either 80 μM 2,4,5‐T or 30 μM 2,4,5‐TCP (Fig. 4). The dechlorination of 2,4,5‐TCP to 3,4‐DCP started after 4 days regardless of the presence of BrES and was only slightly retarded by BrES as previously reported for several tetrachloroethene‐dechlorinating enrichment cultures (Löffler *et al*., 1997). However, the ether cleavage of 2,4,5‐T was completely inhibited. Qualitative analysis of the gas headspace after 8 days showed accumulation of methane in cultures without BrES but no methane in cultures supplemented with BrES. Together, these findings suggest a role for methanogens in the initial attack of 2,4,5‐T.

### Hydrogen is the electron donor for 2,4,5‐T ether cleavage and reductive dechlorination of 2,4,5‐TCP

**Figure 5:**
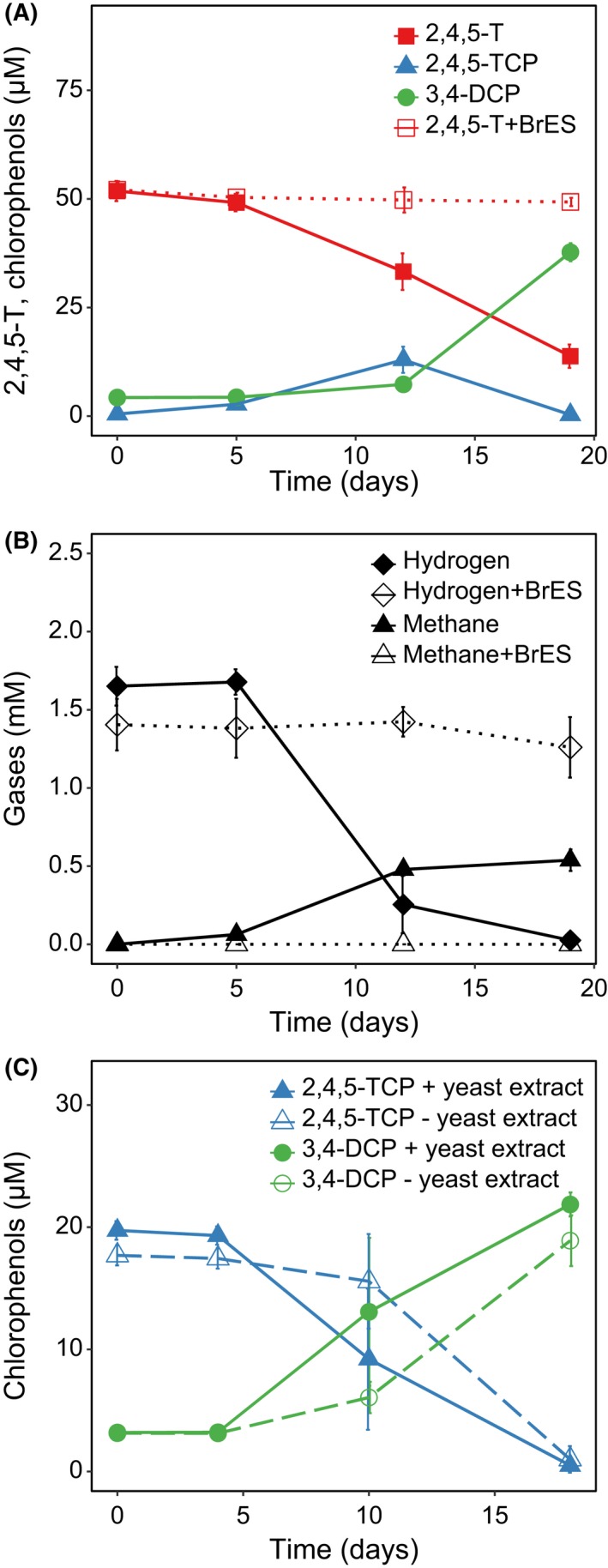
Transformation of 2,4,5‐T (A) and 2,4,5‐TCP (C) with hydrogen as sole electron donor. Titanium (III) citrate was substituted by titanium (III) nitrilotriacetate as reducing agent. Yeast extract was omitted except for one culture variant (see below). B. Hydrogen consumption and methane formation during 2,4,5‐T transformation. Hydrogen was added to the headspace resulting in a nominal concentration of 1.5 mM corresponding to a dissolved concentration of 36 μM. Cultures supplemented with 5 mM bromoethane sulphonate served as negative control for 2,4,5‐T ether cleavage and methane production. Shown are mean values and SD from triplicate (A, C) and duplicate (B) cultures.

Because 2,4,5‐T degradation started only after complete fermentation of pyruvate and when hydrogen appeared in the cultures (Fig. 3A and B), we hypothesized that hydrogen might be the electron donor for both processes, i.e. 2,4,5‐T ether cleavage and reductive dechlorination of 2,4,5‐TCP. To test this, all potential electron donors except hydrogen were excluded from the medium: titanium citrate was substituted by 1 mM titanium nitrilotriacetate and both, pyruvate and yeast extract were omitted. Hydrogen was added to the headspace to provide a nominal concentration of 1.5 mM. Ether cleavage of 2,4,5‐T started at day 5 concomitant with the decrease in hydrogen and was accompanied by 2,4,5‐TCP dechlorination from day 12 on (Fig. 5A and B). Thus, transformation of 2,4,5‐T to 3,4‐DCP occurred at a similar rate as with supplementation of pyruvate and citrate (compare Fig. 3A). The main hydrogen consumption process, however, was methanogenesis leading to the formation of 0.5 mM methane (Fig 5B). No ether cleavage and no methanogenesis occurred in the presence of 5 mM BrES, confirming the results obtained in the presence of pyruvate.

### Metaproteome analysis reveals the involvement of specific reductive dehalogenases in 2,4,5‐T transformation and synthesis of methanogenesis proteins

Label‐free shotgun metaproteomics was applied to identify the protein core of the enrichment culture BH1.1/pyruvate in the presence and absence of 2,4,5‐T. Therefore, cultures were incubated with and without 2,4,5‐T. To obtain a more detailed insight into the augmenting effect of yeast extract (e.g. Fig. 5C), both sets of cultures were cultivated either with (−/+YE) or without yeast extract (−/−YE), corresponding to the conditions described for the 16S rRNA gene analysis above. The cultures were harvested after 11 days, when one‐third (−/+YE) and two‐thirds (−/−YE) of the 2,4,5‐T were converted to 3,4‐DCP. In the absence of yeast extract, the total number of identified protein groups was *n* = 3022 and *n* = 3010 with and without 2,4,5‐T, respectively, whereas these identifications were about 27% higher in the presence of yeast extract (*n* = 3820 and *n* = 3868) (Table S3).

A principal component analysis (PCA) separated the samples into four groups, corresponding to the four growth conditions (Fig. S6). The presence or absence of yeast extract accounted for a high proportion of total variability in this data (35.3%). The second principal component accounted for 7.8% of the total variability and discriminated the cultures further into those exposed and those not exposed to 2,4,5‐T.

**Figure 6:**
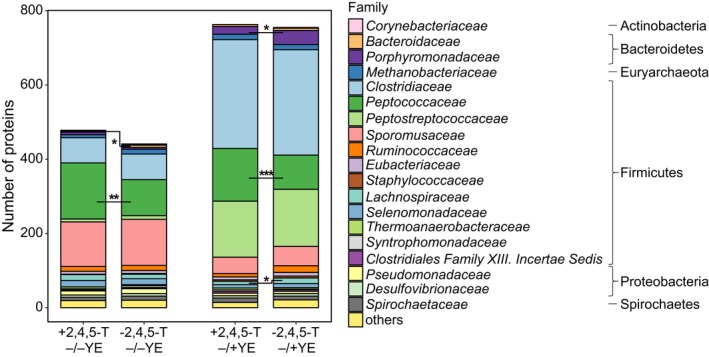
Phylogenetic distribution of proteins. Abundances calculated from the number of proteins detected for each family in the enrichment cultures using different cultivation conditions. Cultures were supplemented with 2,4,5‐T (columns 1 and 3) and without (columns 2 and 4). One set of cultures each was incubated with yeast extract (YE) (columns 3, 4) and without (columns 1, 2). Asterisks indicate significant differences between +2,4,5‐T and ‐2,4,5‐T (*, *P* < 0.05; **, *P* < 0.01; ***, *P* < 0.001). All families with an abundance <1% were summarized in ‘others’. 71% of all proteins could not be classified until family level and are excluded from the figure.

Because of the high complexity of the enrichment culture, about 35% of all identified protein groups could not be assigned to distinct phyla and are therefore named as *heterogeneous;* another 10% could not be assigned. The highest number of protein groups was assigned to the phylum *Firmicutes* (approx. 50%), whereas only 1% or less represented proteins from *Bacteriodetes*,* Proteobacteria, Actinobacteria*,* Spirochaetes*,* Synergistetes, Chloroflexi* or *Euryarchaeota* (Fig. S7). Only the *Firmicutes* were significantly (*P* = 0.02) more abundant in 2,4,5‐T cultures without yeast extract. On the family level, *Peptococcaceae* showed a significant (p_−/−YE_ = 0.001, p_−/+YE_ = 0.0002) increase in protein numbers after 2,4,5‐T addition under both conditions (Fig. 6). *Peptococcaceae* were represented by the genera *Desulfitobacterium*,* Desulfotomaculum* and *Desulfosporosinus*. Interestingly, the numbers of all three genera responded positively to the presence of 2,4,5‐T. Moreover, the number of quantified *Desulfitobacterium* protein groups doubled in the presence of 2,4,5‐T (from *n* = 29 to *n* = 68 and from *n* = 16 to *n* = 32 in the absence and presence of yeast extract, respectively, p_−/−YE_ = 0.003, p_−/+YE_ = 0.0002, Table S3), which indicates the growth advantage gained by the organohalide‐respiring bacterium.

Comparative metaproteome analysis revealed significant changes depending on the presence of 2,4,5‐T. All protein groups with a log2‐fold change ≥ 1 due to the presence of 2,4,5‐T and the protein groups unique to the cultures that were grown with 2,4,5‐T with an abundance above the median of all protein groups are listed in Table S4. From *n* = 60 protein groups with an increase in abundance level (as indicated by the Benjamini–Hochberg adjusted *P* < 0.05), only three (B8FVX4, A0A098AWR7 and A0A098BB7J4) increased under both conditions, with and without yeast extract (Table 2). However, nine protein groups showed clearly higher protein abundance under both conditions as indicated by their unique presence in 2,4,5‐T‐supplemented cultures or a significant positive fold change (raw *P* < 0.05, Table 2). They included an alcohol dehydrogenase, a formylmethanofuran dehydrogenase subunit, an electron‐transfer flavoprotein, ABC transporters and an uroporphyrin‐III C‐methyltransferase (Table 2). Eight protein groups more highly abundant in the presence of 2,4,5‐T under either one or both conditions were assigned to *Desulfitbacterium* (Fig. S8A, B and Table S4). Interestingly, the highest protein abundance difference (18.3‐fold, adj. *P* = 0.009) was observed in the presence of yeast extract assigned to reductive dehalogenases (B8FVX4). Sequence alignment indicated that the assigned protein is the reductive dehalogenase RdhA3 of *D. hafniense* strain DCB‐2^T^ (B8FVX4) or the almost identical CprA5 of *D. hafniense* strain PCP‐1 (Q6V7J3, Table 3). Both are able to dehalogenate 2,4,5‐trichlorophenol (Bisaillon *et al*., 2010; Mac Nelly *et al*., 2014). Without yeast extract, this protein group was unique to the culture with 2,4,5‐T (*P* = 0.02). In the presence of 2,4,5‐T, it reached an abundance level above the median of all proteins, with or without YE. Only one additional reductive dehalogenase was detected in the metaproteome data, which showed high similarity to a putative reductive dehalogenase (Q8RPG3, *D. hafniense* strain DCB‐2^T^) and to the pentachlorophenol reductive dehalogenase CprA3 (Q9ANS1, *D. hafniense* strain PCP‐1). CprA3 is not able to dechlorinate 2,4,5‐TCP (Bisaillon *et al*., 2010). Still, this protein group was only detected when cells were cultivated with 2,4,5‐T, irrespective of whether yeast extract was present in the medium (*P* > 0.05), but its abundance was below the median of all protein groups and approximately 10‐fold lower than RdhA3/CprA5.

**Table 2:** Protein groups upregulated at least twofold by 2,4,5‐T (adj. *P* < 0.05) or unique in the presence of 2,4,5‐T under both conditions, with and without yeast extract.^a^

Accession no. of meta‐protein	Description of lowest common ancestor	Taxon of lowest common ancestor	log2 fold change	
			Without YE	With YE
			+ 2,4,5‐T versus – 2,4,5‐T	+ 2,4,5‐T versus – 2,4,5‐T
B8FVX4	Reductive dehalogenase	*Peptococcaceae*	Unique for 245T^b^	4.2
Q9ANS1	Reductive dehalogenase	*Desulfitobacterium*	Unique for 245T^c^	Unique for 245T^c^
518058910	ABC‐type oligopeptide transport system, periplasmic component	*Sedimentibacter* sp. B4	Unique for 245T^c^	Unique for 245T^d^
A0A098AWR7	Alcohol dehydrogenase, class IV	*Clostridiales*	3.0	3.1
A0A098B7J4	Uncharacterized protein conserved in bacteria	*Desulfitobacterium hafniense*	1.9	2.7
A0A098AW87	Formylmethanofuran dehydrogenase subunit E	*Clostridiales*	2.5	2.7^e^
G9XMD7	Electron‐transfer flavoprotein, beta subunit	*Clostridiales*	1.5^e^	1.9
A0A098AV40	ABC‐type Fe3 + ‐hydroxamate transport system, periplasmic component	*Desulfitobacterium*	2.7^e^	2.5
A4J9D6	Dissimilatory sulphite reductase (desulfoviridin), alpha and beta subunits	Heterogeneous	2.7^e^	1.6
Q24S74	Putative cell wall‐binding domain	*Desulfitobacterium*	1.3^e^	1.1
B8FVX9	Uroporphyrinogen‐III methylase	Heterogeneous	1.4^e^	1.9
A0A098AWK9	ABC‐type dipeptide transport system, periplasmic component	*Firmicutes*	1.7^e^	1.6

**a**. For a complete list of proteins upregulated by 2,4,5‐T in the presence and absence of yeast extract or in only one condition see Table S4. YE, yeast extract; 2,4,5‐T, 2,4,5‐trichlorophenoxyacetic acid;.

**b**. Protein abundance >median of all proteins in this condition.

**c.** Protein abundance <median of all proteins in this condition.

**d**. Protein abundance <median‐1SD (standard deviation) of all proteins in this condition.

**e**. Adjusted *P*‐value >0.05, raw *P*‐value <0.05.

**Table 3:** Reductive dehalogenase proteins and the number of identified peptides in two different cultivation experiments

Accession no. of meta‐protein	Accession	Gene name	Description	Reference^a^	∑ Protein coverage	AAs	Taxon	Peptides					
								1^st^ experiment^b^				2^nd^ experiment^c^	
								− YE + 2,4,5‐T	− YE ‐ 2,4,5‐T	+ YE + 2,4,5‐T	+ YE ‐ 2,4,5‐T	− YE + 2,4,5‐T 7 days	− YE + 2,4,5‐T 14 days
B8FVX4^b^/Q6V7J3^c^	B8FVX4	*rdhA3*, Dhaf_0696	Reductive dehalogenase	(Mac Nelly *et al*., 2014)	40.4^b^ 23.1^c^	550	*Desulfitobacterium hafniense* strain DCB‐2^T^	15	–	14	2	–	9
	Q6V7J3	*cprA5*	3,5‐dichlorophenol reductive dehalogenase	(Thibodeau *et al*., 2004)	38.7^b^ 23.2^c^	548	*Desulfitobacterium hafniense* strain PCP‐1	14	–	13	1	–	9
Q9ANS1	Q8RPG3	*rdhA5,* Dhaf_0713	Putative reductive dehalogenase	(Kruse *et al*., 2017)	22.3^b^	458	*D. hafniense* strain DCB‐2^T^	6	–	5	–	–	–
	Q9ANS1	*cprA3*	CprA‐like protein (Fragment)	(Bisaillon *et al*., 2010)	26.8^b^	380	*D. hafniense* strain PCP‐1	6	–	5	–	–	–

AA, number of amino acids; YE, yeast extract; 2,4,5‐T, 2,4,5‐trichlorophenoxyacetic acid.

**a**. Reference for the functional description of protein.

**b**. First experiment (cultivation with and without 2,4,5‐T, both, in the presence and absence of yeast extract.

**c**. Second experiment (two sequential samples from cultivation with 2,4,5‐T and without yeast extract).

The 27 quantified protein groups assigned to *Euryarchaeota* did not show a 2,4,5‐T‐dependent synthesis except for a subunit of the coenzyme F420‐reducing hydrogenase in the presence of yeast extract (Fig. S8, Table S5). However, several protein groups directly involved in methanogenesis and mostly assigned to the *Methanobacteriaceae,* such as 5,10‐methylene tetrahydromethanopterin reductase, subunits of the methyl coenzyme M reductase and tetrahydromethanopterin S‐methyltransferase were present under all tested conditions, confirming the observed methane production.

The high presence of the reductive dehalogenase RdhA3/CprA5 was further investigated in a separate set of cultures grown without yeast extract (−/− YE) and with 80 μM of 2,4,5‐T. Ether cleavage was already indicated after 7 days by the accumulation of 22 μM 2,4,5‐TCP, which only started to be subject to dechlorination (5 μM 3,4‐DCP was formed). After 14 days, 23 μM 3,4‐DCP had formed indicating the progress of dechlorination (Fig. S9). Metaproteomics was performed at both time points from triplicate cultures (Table S6). While after 7 days, no reductive dehalogenase was identified, after 14 days, RdhA3/CprA5 was identified as the only reductive dehalogenase, with protein abundance above the median of all protein groups. This indicates an onset of synthesis when 2,4,5‐TCP started to accumulate in the cultures (Table 3 and Table S6). The *Euryarchaeota* did not show a differential protein pattern over time, which could be related to the observed ether cleavage activity.

## Discussion

### Transformation reactions in 2,4,5‐T‐degrading enrichment cultures

**Figure 7:**
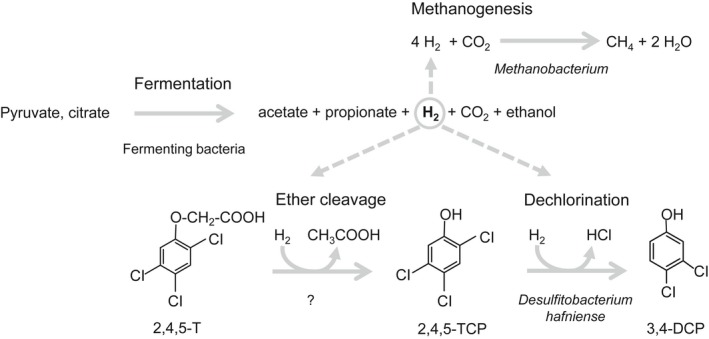
Proposed interaction scheme of members of the BH1.1 microbial community in the conversion of 2,4,5‐trichlorophenoxyacetic acid (2,4,5‐T) via 2,4,5‐trichlorophenol (2,4,5‐TCP) to 3,4‐dichlorophenol (3,4‐DCP). The hydrogen formed from the fermentation of organic acids allows growth of hydrogenotrophic methanogens and is the electron donor for ether cleavage of 2,4,5‐T and for reductive dechlorination. Whereas the ether‐cleaving organism is not known yet, *Desulfitobacterium hafniense* uses 2,4,5‐TCP as the terminal electron acceptor in organohalide respiration. Full arrows: observed reactions; dashed arrows point to the hydrogen consumption processes.

Three strictly anaerobic, 2,4,5‐T‐degrading enrichment cultures were studied, originating from different sites of an active landfill involved in treating Agent Orange‐contaminated soil. Although the primary enrichment cultures showed a broader spectrum of products (Dinh *et al*., 2015; see also the Description of primary and secondary enrichment cultures in the Supporting Information), all enriched cultures clearly showed the transformation of 2,4,5‐T via the initial cleavage of the side‐chain to 2,4,5‐TCP and a subsequent dehalogenation to 3,4‐DCP as the final product (Fig. 7). Cleavage of the ether bond of phenoxyacetic acid‐based herbicides such as 2,4,5‐T or 2,4‐D as the initial degradation reaction was previously reported for microcosms from estuarine sediments (Eder, 1980), sewage sludge (Mikesell and Boyd, 1984; Gibson and Suflita, 1986) and a sandy aquifer (Arildskov *et al*., 2001), and in most instances, it was followed by dehalogenation of the formed chlorophenols to less chlorinated phenols. However, the sequence of ether cleavage and dehalogenation can also be reversed, as reported for a methanogenic aquifer. In this instance, the parent molecule 2,4,5‐T was dehalogenated to 2,5‐ and 2,4‐D, followed by different orders of dehalogenation and ether cleavage reactions resulting in a broad spectrum of products (Gibson and Suflita, 1990). It is likely that the order of transformation reactions is influenced by specific environmental or culture conditions. For instance, cultures pre‐adapted to dehalogenation of chlorophenols also started with the dehalogenation of 2,4,5‐T followed by the cleavage of the side‐chain (Bryant, 1992). On the other hand, cultures enriched with phenoxyacetic acid as the sole carbon source were able to cleave the side‐chains of mono‐ to trichlorinated phenoxyacetic acids but were unable to dehalogenate the chlorophenols that were formed (Gibson and Suflita, 1993). The ether‐cleaving activity in these cultures was higher for non‐chlorinated and mono‐ and dichlorinated phenoxyacetic acids and was lowest for 2,4,5‐T. This was completely different from the substrate specificity observed for our enrichment culture, which had a preference for 2,4,5‐T and 2,4‐D compared with the non‐chlorinated phenoxyacetate. This suggests that possibly the 2,4,5‐T and 2,4‐D contamination in the active landfill in Bien Hoa, together with our enrichment strategy, selected for microorganisms with specific ether‐cleaving activities for chlorinated phenoxyacetic acids. The existence of diverse combinations of degradation reactions supports the hypothesis that not one single bacterium is responsible for the complete degradation sequence but rather an interacting community combines to effect the two different types of reactions, i.e. the ether cleavage and the dehalogenation. However, neither the structure nor single members of these communities have been described so far.

### Central role of hydrogen in the metabolic processes of the microbial community

As our intention was to simulate the supplementation of the treatment plant with organic, fermentable material, we added selected organic acids to enrich for anaerobic 2,4,5‐T degradation. As indicated by 16S rRNA gene amplicon sequencing and metaproteomics of the enrichment culture BH1.1, a complex community of anaerobic organisms was established, including fermenting bacteria and methanogens. Their metabolic activities were recorded and led to the identification of hydrogen as a central intermediate (Fig. 7, see also Fig. S4 and the explanations in the legend). Pyruvate was fermented to acetate, propionate and CO_2_ using the propionic acid fermentation pathway. Based on the determined propionic acid concentration and the stoichiometry of this reaction (Fig. S4, equation a), the major portion (3.6 mM) of the total added pyruvate was converted in this way. Key enzymes of the propionic acid fermentation pathway (pyruvate/oxaloacetate carboxyltransferase, methylmalonyl‐CoA mutase) were highly abundant in the metaproteome (Table S3). In addition, pyruvate:ferredoxin oxidoreductase and pyruvate formate lyase were abundant, suggesting that the remaining pyruvate (0.6 mM) was oxidized or cleaved to acetyl‐CoA with concomitant formation of either reduced ferredoxin or formate respectively. Both of these products can deliver hydrogen by the action of hydrogenases (reduced ferredoxin) or the formate hydrogen lyase complex (formate). Based on these assumptions, approximately 0.6 mM hydrogen could be formed from the fermentation of the remaining pyruvate (Fig. S4, equation b).

The subsequent fermentation of citrate should yield acetate, CO_2_, ethanol and hydrogen in a distinct ratio (Walther *et al*., 1977). Based on the reaction stoichiometry, the 1.8 mM citrate initially present could result in the formation of 0.7 mM hydrogen (Fig. S4, equation c). Thus, in total, a concentration of ~1.3 mM hydrogen might have been available in the cultures. Interestingly, for the formation of the detected amount of methane (0.31 mM at day 8), 1.25 mM hydrogen is theoretically required (Fig. S4, equation d). This explains why hydrogen was already close to the detection limit at day 8, when fermentation processes and methanogenesis ceased (Fig. 3A and B). Acetogenesis and ethanol oxidation might exert an additional limited influence on the total hydrogen pool. Both processes are restricted to distinct hydrogen concentrations. Hydrogen consumption by acetogenic bacteria requires > 520 ppmV hydrogen (Cord‐Ruwisch *et al*., 1988), corresponding to a nominal concentration of 20 μM in our cultures, which is only available around day 4. On the other hand, hydrogen formation from the oxidation of ethanol is energetically feasible only at very low hydrogen partial pressures, which in our cultures probably occurred only after day 8, when the concentration of the oxidation product acetate marginally increased (Fig. 3B, Fig. S3B).

In a separate experiment (Fig. 5), we could show that hydrogen served as sole electron donor for 2,4,5‐T ether cleavage and dechlorination of 2,4,5‐TCP (Fig. 7). Because of the low concentration of 2,4,5‐T, micromolar concentrations of hydrogen were sufficient for the conversion of 2,4,5‐T. In pyruvate‐ plus citrate‐supplemented cultures, 2,4,5‐T ether cleavage and dechlorination proceeded, even when hydrogen dropped below the detection limit of 100 ppmV (corresponding to ~4 μM nominal concentration). For the reductive dechlorination step, this can be explained by the fact that organohalide respiration is energetically feasible at H_2_ concentrations as low as 0.4 ppmV (Löffler *et al*., 1999).

### The ether cleavage of 2,4,5‐T can be uncoupled from the subsequent reductive dechlorination of 2,4,5‐TCP

For the Bien Hoa cultures, we obtained evidence that at least two organisms are involved in the sequential degradation of 2,4,5‐T (Fig. 7). Both steps, i.e. ether cleavage and 2,4,5‐TCP dechlorination, could be inhibited individually while preserving the other step. This was achieved by either BrES addition (Fig. 4) or by lactate addition instead of pyruvate. Subcultures that lost the 2,4,5‐TCP dehalogenation activity (Fig. 1, Table S2) continued to cleave the 2,4,5‐T ether bond, even in repeated subcultures with lactate. Aryl ether linkages are important structural elements of the abundant and recalcitrant lignin molecule (Lewis and Yamamoto, 1990). Cleavage of the ether bonds might enable bacteria to access the guaiacyl‐ or syringyl‐derived monomers as sources of carbon or reducing equivalents. Whereas aerobic bacteria make use of so‐called β‐etherases of the glutathione‐S‐transferase superfamily (Gall *et al*., 2014), a different type of aryl ether‐cleaving mechanism was described for anaerobic bacteria. Acetogenic bacteria and interestingly members of the non‐acetogenic genus *Desulfitobacterium* possess aryl‐alkyl‐ether‐cleaving methyl transferases, so‐called O‐demethylases, which transfer the O‐methyl group from monomers of the lignin molecule such as guaiacol, vanillate or veratrol to tetrahydrofolate. The methyl group is finally oxidized to CO_2_ by acetogenesis. The resulting reducing equivalents allow growth using terminal electron acceptors such as fumarate (Kreher *et al*., 2010; Studenik *et al*., 2012). Despite the presence of several O‐demethylases encoded in the genomes of *Desulfitobacterium* (Nonaka *et al*., 2006; Kim *et al*., 2012), none of these proteins was detected in the metaproteome (Tables S3 and S6), strongly suggesting that they do not play a role in 2,4,5‐T ether cleavage.

The inhibitory effect of BrES on 2,4,5‐T ether cleavage and methanogenesis suggested a role of methanogens in the first 2,4,5‐T transformation step (Sparling and Daniels, 1987). Metaproteomics revealed proteins directly involved in methanogenesis by *Methanobacterium* and *Methanosarcina* species, both known to be hydrogenotrophs. The relatively slow, rate‐limiting ether cleavage and the absence of a 2,4,5‐T‐dependent upregulation of methanogenic proteins (Table S5) could point to a side reaction of a known enzyme, rather than a specific reaction of an unknown enzyme of methanogens. In addition, key membrane proteins might have escaped detection during our analyses, because the proteomic analysis of membrane proteins is challenging. A membrane‐associated 2,4,5‐T ether cleavage is conceivable as the formed product 2,4,5‐TCP is rather toxic (Madsen and Aamand, 1992). Hence, its release outside the cell might be beneficial. However, to date, we cannot exclude that BrES might also inhibit the bacterial community non‐specifically, as was described for several non‐methanogenic dechlorinating cultures (Löffler *et al*., 1997). The mode of this inhibition has not been resolved; however, we detected possible target proteins for this inhibition, the synthesis of which is strongly upregulated in the presence of 2,4,5‐T (Table 2). These proteins, including an alcohol dehydrogenase, are mostly of heterogeneous origin within the *Clostridiales* and are also encoded in *Desulfitobacterium* genomes. We tested the *D. hafniense* strains DCB‐2^T^ and TCP‐A for their capability to transform 2,4,5‐T. Whereas both dechlorinated 2,4,5‐TCP to 3,4‐DCP, they were unable to attack 2,4,5‐T (data not shown), ruling out their involvement in 2,4,5‐T ether cleavage.

### Desulfitobacterium hafniense *catalyses the reductive dechlorination of 2,4,5‐TCP*

Our data strongly support that *Desulfitobacterium hafniense* is the 2,4,5‐TCP‐dehalogenating organism (Fig. 7). Both the 16S rRNA gene amplicon sequences and the metaproteome data indicated the presence of *D. hafniense* as the only representative of known organohalide‐respiring bacteria (Fig. S5). Its association with the dechlorination process was indirectly shown by PCR with genus‐specific primers, where PCR products were exclusively obtained for cultures showing dehalogenating activity (Fig. S2). Consequently, the loss of the dehalogenation capability in lactate‐supplemented cultures can be explained by the inability of *Desulfitobacterium* to grow by lactate fermentation (Utkin *et al*., 1994; Christiansen and Ahring, 1996). In contrast to pyruvate‐supplemented cultures, *D. hafniense* supplemented with lactate was most likely unable to proliferate during the initial fermentation phase. In addition, fermentation of lactate via the canonical propionic acid or acryloyl‐CoA pathways does not yield hydrogen. Consequently, the source of hydrogen might have been restricted to citrate in these cultures. The lower amount of hydrogen and the toxicity of the accumulating 2,4,5‐TCP might have impeded the fast and productive start of organohalide respiration and supported the out‐dilution of *Desulfitobacterium* during successive transfers.

The increase in *Desulfitobacterium* 16S rRNA genes along with reductive dehalogenation activity (Fig. 2) is in accord with the organohalide respiration capacity of this organism. Notably, the synthesis of two reductive dehalogenases of *D. hafniense* in response to 2,4,5‐T was indicated by metaproteome analyses. An orthologue of RdhA3 from strain DCB‐2^T^ had overall the highest protein abundance, which was almost 20‐fold higher in the presence of 2,4,5‐T (−/+YE), and was produced as soon as 2,4,5‐TCP started to accumulate (Table 2). RdhA3 and its orthologue CprA5 in *D. hafniense* strains DCB‐2^T^ and PCP‐1 were described to be inducible by 3,5‐dichlorophenol (Gauthier *et al*., 2006; Bisaillon *et al*., 2011; Mac Nelly *et al*., 2014). Both enzymes dehalogenated 3,5‐DCP at the *meta*‐position and exhibited highest activity with this compound but they also accepted 2,4,5‐TCP as substrate. The dehalogenating activity of RdhA3 and CprA5 on 2,4,5‐TCP was one‐third and one half of the activity on 3,5‐dichlorophenol respectively (Thibodeau *et al*., 2004; Mac Nelly *et al*., 2014). In these studies, 2,4,5‐TCP was dechlorinated at the *ortho* position leading to 3,4‐DCP as the final product, as was observed in our enrichment cultures. In addition, both enzymes can dechlorinate 2,4‐DCP (the degradation product of 2,4‐D) at the *ortho* position, exhibiting an activity only slightly lower than with 3,5‐DCP. Consequently, having an enzyme with these substrate specificities provides a selective advantage for *Desulfitobacterium* in our enrichment cultures. The other detected reductive dehalogenase is an orthologue of Dhaf_0713 or the identical CprA3 of *D. hafniense* strains DCB‐2^T^ and PCP‐1 respectively (Table 3). The *cprA3* gene was shown to be transcriptionally induced by 2,4,6‐trichlorophenol (Bisaillon *et al*., 2011) but was also transcribed in the absence of chlorophenols (Gauthier *et al*., 2006). Interestingly, the purified CprA3 of strain PCP‐1 dechlorinated highly chlorinated phenols such as penta‐ and tetrachlorophenols and some trichlorophenols, but not 2,4,5‐TCP (Bisaillon *et al*., 2010). This, together with the low amount of the corresponding dehalogenase in the metaproteome data, suggests an unspecific synthesis of this dehalogenase.

Interestingly, two other *Desulfitobacterium* proteins showed a similar response to 2,4,5‐T as that observed for RdhA3, which were electron‐transfer flavoproteins (16‐fold higher protein abundance, Table S4). An orthologue (Desde_3368) of one of these flavoproteins (A0A098B093, 93% amino acid identity) was recently described to be more highly abundant in the proteome of *D. dehalogenans* during organohalide respiration with 3‐chloro‐4‐hydroxyphenylacetate, along with the respective reductive dehalogenase CprA, suggesting a specific function in the respiration process (Kruse *et al*., 2015). The high level of upregulation of RdhA3 and these flavoproteins points to their chlorophenol‐dependent induction, whereas the lower abundance changes in other *Desulfitobacterium* proteins might reflect the general growth advantage of the strain when using organohalide respiration (Table 2, Table S4).

## Conclusion

The addition of fermentable organic material to the bioremediation plant in Vietnam was a highly beneficial measure, allowing a complex organohalide‐degrading community to become established including *Desulfitobacterium*, a well‐known chlorophenol‐dehalogenating bacterium. In the primary microcosms, additional degradation products of 2,4,5‐T, such as 2,5‐dichlorophenol and 3‐chlorophenol, were observed. In the soil samples, members of the *Dehalococcoidia* as well as *Desulfitobacterium* were detected by PCR‐based approaches (Dinh *et al*., 2015). This finding suggests that an even more complex microbial 2,4,5‐T‐degrading network within the active landfill in Bien Hoa exists, from which we have investigated only a subset of existing reactions. The long history of 2,4,5‐T and 2,4‐D contamination of the treated soils together with the treatment strategy likely enabled the favourable enrichment of biodegrading communities. However, our data demonstrate that disturbance of this interaction, loss of members of the community or limitation of electron donors slows down the transformation process or results in altered end‐products. Some of these might be more toxic and persistent, such as 2,4,5‐TCP. Therefore, a detailed understanding of the total microbial degradation process is urgently required. Understanding the molecular basis of 2,4,5‐T degradation is not only important with respect to the treatment process in Bien Hoa but also with regard to the present‐day use of phenoxycarbonic acid herbicides in agriculture and will aid in reducing the associated risk of groundwater contamination worldwide.

## Experimental procedures

### Chemicals

All chemicals were of the highest quality available. 2,4,5‐T and the other phenoxyacetic acid derivatives were obtained from Dr. Ehrenstorfer (Augsburg, Germany), Sigma‐Aldrich (Steinheim, Germany) or Acros Organics (Geel, Belgium). The 2‐, 3‐ and 4‐monochlorophenol (MCP) derivatives were obtained from Laborchemie (Apolda, Germany), Merck (Darmstadt, Germany) and Jenapharm (Jena, Germany) respectively. The dichlorophenols (DCP) were obtained from Applichem (Darmstadt, Germany), Merck and Dr. Ehrenstorfer. Deuterated standards and pentafluorobenzylbromide were purchased from Sigma, and *N‐*methyl*‐N‐*trimethylsilyl‐heptafluorobutyramide (MSHFBA) was purchased from Macherey‐Nagel (Düren, Germany).

### Origin of samples, enrichment cultures and sampling

The primary enrichment cultures were established from a bioactive landfill constructed on the site of a former military airbase in Bien Hoa in Dong Nai Province. For details of the sampling location, see the Description of the bioactive landfill in the Supporting Information.

Anoxic mineral medium ‘204’ (Bunge *et al*., 2008) was prepared and supplemented with 0.005% (w/v) yeast extract, acetate (2.5 mM) as a potential carbon source and a mixture of pyruvate and lactate (5 mM each) as potential electron donors. Titanium (III) citrate (0.4 mM) plus FeS (0.15 mM) (McCue *et al*., 1996) or titanium (III) citrate (1 mM) alone was added as reducing agents. As monitored with a redox electrode in the beginning and at the end of the experiment, both reducing agents resulted in redox potentials below – 350 mV throughout the experiments. Where indicated, titanium (III) citrate was substituted by titanium (III) nitrilotriacetate. A stock solution of 2,4,5‐T (5 mM in acetone) was initially added separately to empty sterile 50‐mL serum bottles to give a final concentration of 75–100 μM. Acetone was evaporated by a stream of filter‐sterilized N_2_ gas before the sterile anoxic medium was added. Later subcultures were supplied more conveniently with 2,4,5‐T by adding an appropriate volume of an anoxic aqueous 0.9 mM stock solution (water solubility of 2,4,5‐T is 1.09 mM at 20°C) before medium sterilization. Subcultures were established in duplicate, triplicate or quadruplicate by transferring 10% (vol/vol) into fresh medium. The bottles were closed with Teflon‐coated rubber stoppers, and sterile anoxic medium was added to a volume of 20–40 ml. The gas headspace of the serum bottles was flushed with a filter‐sterilized CO_2_/N_2_ gas mixture (20%/80%, vol/vol). Duplicate cultures without inoculum served as abiotic controls. For the analysis of 2,4,5‐TCP dechlorination, an anoxic stock solution (10 mM dissolved in 0.01 N NaOH) was added to the medium at a concentration of 30–40 μM. Alternative phenoxyacetic acids were also added from 5 mM stock solutions in acetone as described above for 2,4,5‐T. Where indicated, 5 mM 2‐bromoethane sulphonic acid (BrES) was added from a 0.4 M stock solution.

Starting from the first subculture obtained from the primary enrichment from sampling sites BH1.1, BH4.3 and BH4.4 (Dinh *et al*., 2015), the cultures were transferred several times in periods of 3–6 months and incubated with 100 μM 2,4,5‐T and 5 mM each of pyruvate and lactate statically at 30°C. Table S1 shows a more detailed description of the enrichment procedure. In an attempt to enrich the 2,4,5‐T‐degrading bacteria by reducing the amount of fermentable substrates, starting with the 5^th^ transfer, two separate cultivation lines were established for every sample, which received either 2.5 mM pyruvate or 2.5 mM lactate. In later subcultures, the pyruvate or lactate concentration was again increased to 5 mM and acetate was omitted. Samples (0.6–2 ml) were withdrawn, centrifuged (10 min, 6000–12 000 × *g*) prior to gas chromatography (GC) or high pressure liquid chromatography (HPLC) for subsequent analyses of 2,4,5‐T, chlorophenols and organic acids. The supernatants were stored at 4°C prior to analysis. For DNA extraction, a two‐step centrifugation was carried out at 4°C to separate first the black particulate FeS before the cells were precipitated (1500 × *g*, 4 min; subsequently, the supernatant was again subjected to centrifugation at 16089 × *g* for 15 min). The cell pellet was stored frozen at −20°C until DNA extraction was performed. For gas chromatography–mass spectrometry (GC‐MS) analyses, 10 ml samples were taken and stored at −20°C until analysis. For proteome analyses, 20 ml of cultures was harvested by centrifugation (2520 × *g*, 30 min, 8°C) and the cell pellet was stored at −80°C until analysis.

### Analytical techniques

2,4,5‐T and the chlorophenols formed were analysed by HPLC (Elite LaChrom, VWR‐Hitachi) using a C18‐column (LiChrospher, 250 × 4 mm, 5 μm), an oven temperature of 30°C and a flow rate of 1 ml min^−1^. 2,4,5‐T, 2,4,5‐TCP and 3,4‐dichlorophenol (DCP) were analysed in a water–acetonitrile gradient and detected at 210 nm as described (Rice *et al*., 2005). The compounds were quantified based on a four‐level calibration curve (12.5–100 μM).

Chlorophenols were also analysed by gas chromatography (GC) using a GC‐2010 instrument (Shimadzu, Japan) equipped with an electron‐capture detector (ECD). Samples (300 μl) of the culture supernatant were acetylated using 600 μl of acetylation buffer (0.025 M NaHCO_3_ adjusted to pH 9.9 by 1 M NaOH) and 60 μl of acetic anhydride. The acetylated chlorophenols were extracted into hexane. 3,4,5‐trichloroveratrole served as internal standard, and the samples were analysed as previously described (Pöritz *et al*., 2015).

Gas chromatography–mass spectrometry (GC‐MS) analyses of chlorinated phenoxyacetic acids and phenols were applied using the following protocol for sample extraction and derivatization. Prior to the enrichment step, the pH of the sample was adjusted to 2 and the solid phase (1 g CHROMABOND^®^ C_18_‐Hydra; Macherey‐Nagel) was preconditioned by washing with 5 ml of acetone, 10 ml of methanol and 10 ml of distilled water at pH 2. Each sample was spiked with a defined amount of surrogate standards 2,4‐DCP‐d3 and 2‐(4‐chloro‐phenoxy)propionic acid to observe losses during the sample preparation. Sample introduction and concentration were carried out under atmospheric pressure. After drying the solid phase under vacuum (500 mbar, 4 h), the enriched analytes were eluted using 5 ml of ethyl acetate.

To detect the chlorophenols, a subset of the extract was spiked with internal standards (phenol‐d5 and 2‐MCP‐d4) and then silylated with *N*‐methyl‐*N*‐trimethylsilylheptafluorobutyramide at 80**°**C for 30 min. The derivatization of the phenoxycarboxylic acids was performed in another subset of the extract following the addition of the internal standards 2,4‐dichlorobenzoic acid and 2,4‐D‐d3 and using 2,3,4,5,6‐pentafluorobenzylbromide (2% w/v in toluene) and triethylamine at 110 **°**C for 1 h. GC–MS was performed using GC HP 6890 coupled with MS HP 5972A (Hewlett Packard) and electron impact (EI) ionization (70 eV) and a SLB‐5 ms capillary column (30 m × 0.25 μm internal diameter × 0.25 μm film thickness, Supelco). The mass spectrometer was operated in the selected ion monitoring (SIM) mode.

Organic acids were analysed by HPLC injecting 20 μl of cell‐free supernatant onto an Aminex HPX‐87H column (Bio‐Rad Laboratories, München, Germany) (300 × 7.8 mm, 9 μm) and using 5 mM H_2_SO_4_ as the eluent, a flow rate of 0.6 ml min^−1^ and UV detection at 210 nm.

For the measurement of hydrogen and methane, 200 μl samples were taken from the gas headspace of the cultures by means of a gas‐tight syringe. Samples were analysed using a GC‐2010 Plus instrument (Shimadzu, Japan) equipped with a thermal conductivity detector, a ShinCarbon ST capillary column (100/120 mesh, 2 m, 1/16 in OD, 1.0 mm ID; Restek, Bad Homburg, Germany) and nitrogen as the carrier gas (13.9 ml min^−1^). The injector, detector and column temperatures were set to 140, 150 and 110 °C respectively. The detection limits for hydrogen and methane were 100 ppmV and 600 ppmV, corresponding to dissolved concentrations of 0.08 and 0.8 μM, respectively, calculated as described (Löffler *et al*., 1999). To relate the formation or consumption of the gases to the transformation of organic acids and of 2,4,5‐T, the nominal concentration of both gases was calculated as the total molar amount (dissolved and gaseous) per bottle divided by the culture volume. To reduce the risk of oxygen infiltration through holes in the septa produced by repeated sampling, headspace analyses with the relatively thick needle of the gas‐tight syringe were performed on three varying subsamples of the five culture replicates.

### Molecular biological methods

For the isolation of total DNA from 2 ml of the anaerobic cultures, the NucleoSpin Tissue kit was used according to the instructions of the manufacturer (Macherey‐Nagel). The DNA was eluted in 50–100 μl water and served as template (1 μl) for polymerase chain reactions (PCR) using the HotStarTaq Polymerase (Qiagen, Hilden, Germany) according to the instructions of the manufacturer and 40 cycles of amplification. The following primers were used to detect *Dehalococcoides*: DET730f/DET1350r (Ballerstedt *et al*., 2004), the latter with one degenerate position to match also members of the Pinellas group of *Dehalocococides mccartyi*;* Dehalobacter*: DRE445f (Ballerstedt *et al*., 2004)/DRE1248r (5′‐GGCTTCGCTTCCGTCTG‐3′); and *Desulfitobacterium*: DES436f (5′‐TGTCTTCAGGGACGAACG‐3′)/DES1027r (5′‐CTCATAGCTCCCCGAAGG‐3′). The latter three primers were designed using the ARB software (Ludwig *et al*., 2004). The annealing temperatures were 53, 52 and 53**°**C respectively. Genomic DNA of *Dehalococcoides mccartyi* DCMB5, *Dehalobacter restrictus* PER‐K23 and *Desulfitobacterium hafniense* TCP‐A was used as the respective positive controls.

Quantitative PCR (qPCR) for the enumeration of *Desulfitobacterium* sp. 16S RNA genes was performed using the QuantiTect SYBR Green PCR kit (Qiagen, Hilden, Germany), the recommended amplification conditions over 42 cycles with an annealing temperature of 65**°**C, the primers Dsb406F and Dsb619R (Smits *et al*., 2004) and the pGEM T‐Easy vector carrying the almost full‐length 16S rRNA gene of *Desulfitobacterium hafniense* DCB‐2^T^ as the external standard. The PCR efficiency was 0.91.

Illumina sequencing of 16S rRNA gene amplicons (variable region V4, positions 515 and 806, according to *E**. **coli*‐numbering) was performed by Molecular Research (MRDNA, Shallowater, TX, USA). Sequencing was performed on a MiSeq following the manufacturer's guidelines. Sequence data were processed using MRDNA analysis pipeline. In summary, sequences were joined, depleted of barcodes, then sequences <150 bp removed, and sequences with ambiguous base calls were removed. Sequences were denoised, operational taxonomic units (OTUs) generated and chimeras removed. OTUs were defined by clustering at 3% divergence (97% similarity). Final OTUs were taxonomically classified using BLASTn against a curated database derived from RDPII and NCBI (http://www.ncbi.nlm.nih.gov, http://rdp.cme.msu.edu).

### Metaproteome analysis

#### Protein extraction

For each cultivation conditions of the BH1.1 cultures, the cells were pelleted at 2520 × *g*, 8°C for 30 min. Proteins were extracted as described (Starke *et al*., 2017). Briefly, cells were suspended in SDS buffer (0.1 M Tris/HCl, pH 6.8, 1.25% w/v SDS, 20 mM dithiothreitol) and disrupted using the FastPrep instrument (MP Biomedicals, Santa Ana, CA, USA) and three cycles of freeze and thaw (freeze in liquid nitrogen, thaw in 40°C water bath). Samples were centrifuged (7800 *g*, 10 min, 4°C), and the supernatant was mixed with an equal volume of phenol solution (10 g ml^−1^) and incubated at room temperature for 60 min. The proteins in the phenol phase were precipitated using 100 mM ammonium acetate in methanol. Dried pellets were resuspended in 20 μl of SDS sample buffer, heated at 90°C for 4 min and separated by 1D‐SDS polyacrylamide gel electrophoresis. Proteins were stained with colloidal Coomassie brilliant blue (Merck). After the gel electrophoresis run, each sample lane was cut, separated in tubes, destained, reduced and alkylated. Finally, the protein lysate was proteolytic cleaved using trypsin (Promega). Extracted peptide lysates were desalted using SOLAμ SPE‐plates (Thermo Fisher). Peptides were dissolved in 0.1% (v/v) formic acid and injected to a liquid chromatography tandem mass spectrometer (nanoLC‐MS/MS).

#### Mass spectrometry analysis

Peptide lysates were separated using a 120 min nonlinear gradient from 3.2 to 40% (v/v) acetonitrile, 0.1% (v/v) formic acid on an analytical column (Acclaim PepMap100, 75 μm inner diameter, 25 cm, C18, Thermo Scientific) in a UHPLC system (Ultimate 3000 RSLCnano; Thermo Fisher Scientific, Idstein, Germany). Mass spectrometry was performed on a Q Exactive HF instrument (Thermo Fisher Scientific, Waltham, MA, USA) coupled with a TriVersa NanoMate (Advion, Ltd., Harlow, UK) source in LC chip coupling mode. The following MS settings were selected: loop count 10, normalized higher‐energy collisional dissociation (HCD) 28%, MS scans in the Orbitrap (resolution 120 000, scan range 350–1550 *m/z*, ion count target 3 × 10^6^, injection time 100 ms), MS/MS scans in the quadrupole (isolation window of 1.4 *m/z*, resolution 15 000, scan range 200–2000 *m/z*) and dynamic exclusion 30 s.

#### Data analysis

The acquired MS spectra were processed using Proteome Discoverer (v1.4; Thermo Scientific). MS/MS spectra were searched with the Sequest HT algorithm against a database generated from the entries of the taxa identified by 16S rRNA gene amplicon sequencing in UniProt or the National Center for Biotechnological Information (NCBI, July 2016), resulting in a database with 5 157 306 protein‐coding sequences. Enzyme specificity was selected as trypsin with up to two missed cleavages allowed using 10 ppm peptide ion tolerance and 0.02 Da MS/MS tolerances. Oxidation (methionine) was selected as a dynamic and carbamidomethylation (cysteine) as a static modification. Only peptides with a false discovery rate (FDR) <1% calculated by Percolator (high confidence) and peptide score XCorr >2.1 were considered as identified. PROPHANE was performed on order to assign the protein groups to their phylogenetic origin (http://www.prophane.de/index.php). The protein abundance was quantified using the average MS1‐Area of the top‐3 peptides. Protein abundances were log2‐transformed and median‐normalized. For statistical analysis, proteins quantifiable in ≥ 50% of replicates were imputed using Prostar (imp4p, 10 iterations, no Lapala, http://www.prostar-proteomics.org). Comparative metaproteome analysis was performed using a two‐sided *t*‐test (equal variance), corrected with the Benjamini–Hochberg method at false discovery rate (FDR) <5%. The significance of unique proteins was determined using the Wilcoxon–Mann–Whitney *U* test at an FDR <5%; the significance of taxonomic differences using a two‐sided *t*‐test (unequal variance).

Quantitative protein data were generated by calculating the mean of the protein abundances of at least two replicates of a condition, subtracting the median of each condition and the global minimum to scale all values to zero. Figures were created using an in‐house R‐script using the packages gplots, ggplot2, ggbiplot, dplyr, miscTools and vegan.

## Conflict of interest

None declared.

## Authors’ contributions

DT, DTTH, HA, SF, MG, SS carried out all experiments with the enrichment cultures including qPCR, chemical analyses and metaproteome analyses. DTCH developed the active landfill and organized sampling campaigns in Bien Hoa. MK identified the degradation products using gas chromatography–mass spectrometry. DTCH, UL, and NJ conceived the study, and UL, DT, NJ, DTTH, MK, MvB drafted the manuscript. All the data presented in this manuscript are original data, and all authors read and approved the final manuscript.

## Supporting information


**Fig. S1.** Formation of 3,4‐dichlorophenol in BH4.4, 9^th^ sub‐culture, supplemented with 2,4,5‐T (A) or 2,4,5‐trichlorophenol (2,4,5‐TCP) (B) and 5 mM pyruvate (mean values of triplicate cultures and SD).
**Fig. S2.** Detection of *Desulfitobacterium* by PCR with genus‐specific primers in the 8^th^ subculture of enrichment cultures BH1.1, BH4.3 and BH4.4 incubated with 100 μM 2,4,5‐T and pyruvate or lactate.
**Fig. S3**. Transformation of 2,4,5‐T (A) and fermentation of pyruvate and citrate (B) in enrichment culture BH1.1 cultivated in the presence of yeast extract.
**Fig. S4.** Reaction stoichiometries and mass balance of pyruvate and citrate fermentation and of methanogenesis in the enrichment culture.
**Fig. S5.** Genus‐level distribution based on amplicon sequencing of 16S rRNA genes.
**Fig. S6.** PCA‐Plot of all detected proteins of the metaproteomic analysis of four replicates and of four different conditions.
**Fig. S7.** Phylogenetic distribution of proteins.
**Fig. S8.** Log2‐fold change and Benjamini‐Hochberg adjusted *P*‐values of all quantifiable and unique meta‐proteins compared for +2,4,5‐T and –2,4,5‐T, without YE (A) and with YE (B).
**Fig. S9.** Transformation of 2,4,5‐T by enrichment culture BH1.1 in the absence of yeast extract.
**Table S1.** Description of the transfers of the 2,4,5‐T enrichment cultures used for the experiments.
**Table S2**. Chlorinated phenoxyacetic acids and phenols (μM) identified and quantified by mass spectrometry after 10 days of cultivation in the 6^th^ subculture of enrichment cultures BH1.1, BH 3.4 and BH4.4 and abiotic controls incubated with 100–200 μM 2,4,5‐T and either 2.5 mM pyruvate or 2.5 mM lactate.
**Table S4**. Protein groups upregulated by 2,4,5‐T (adj. *P* < 0.05) or unique in the presence of 2,4,5‐T (with an abundance >median, *P* < 0.05) with and/or without yeast extract.
**Table S5.** Relative abundance of proteins belonging to *Euryarchaeota*.

**Table S3**. Metaproteomic data of the BH1.1 enrichment culture under four different cultivation conditions (first experiment, cultivation with and without 2,4,5‐T, both, in the presence and absence of yeast extract).

**Table S6**. Metaproteomic data of the BH1.1 enrichment culture (second experiment, two sequential samples from cultivation with 2,4,5‐T and without yeast extract, three biological replicates).
