# Supplementary material for: Desulfitobacterium contributes to the microbial transformation of 2,4,5‐T by methanogenic enrichment cultures from a Vietnamese active landfill
Source: Microb Biotechnol. 2018 Aug 16;11(6):1137–56. doi: 10.1111/1751-7915.13301 (PMC6196390; doi:10.1111/1751-7915.13301)
Supplement: Supplementary file 1 — Fig. S1. Formation of 3,4‐dichlorophenol in BH4.4, 9th sub‐culture, supplemented with 2,4,5‐T (A) or 2,4,5‐trichlorophenol (2,4,5‐TCP) (B) and 5 mM pyruvate (mean values of triplicate cultures and SD). Fig. S2. Detection of Desulfitobacterium by PCR with genus‐specific primers in the 8th subculture of enrichment cultures BH1.1, BH4.3 and BH4.4 incubated with 100 μM 2,4,5‐T and pyruvate or lactate. Fig. S3. Transformation of 2,4,5‐T (A) and fermentation of pyruvate and citrate (B) in enrichment culture BH1.1 cultivated in the presence of yeast extract. Fig. S4. Reaction stoichiometries and mass balance of pyruvate and citrate fermentation and of methanogenesis in the enrichment culture. Fig. S5. Genus‐level distribution based on amplicon sequencing of 16S rRNA genes. Fig. S6. PCA‐Plot of all detected proteins of the metaproteomic analysis of four replicates and of four different conditions. Fig. S7. Phylogenetic distribution of proteins. Fig. S8. Log2‐fold change and Benjamini‐Hochberg adjusted P‐values of all quantifiable and unique meta‐proteins compared for +2,4,5‐T and –2,4,5‐T, without YE (A) and with YE (B). Fig. S9. Transformation of 2,4,5‐T by enrichment culture BH1.1 in the absence of yeast extract. Table S1. Description of the transfers of the 2,4,5‐T enrichment cultures used for the experiments. Table S2. Chlorinated phenoxyacetic acids and phenols (μM) identified and quantified by mass spectrometry after 10 days of cultivation in the 6th subculture of enrichment cultures BH1.1, BH 3.4 and BH4.4 and abiotic controls incubated with 100–200 μM 2,4,5‐T and either 2.5 mM pyruvate or 2.5 mM lactate. Table S4. Protein groups upregulated by 2,4,5‐T (adj. P < 0.05) or unique in the presence of 2,4,5‐T (with an abundance >median, P < 0.05) with and/or without yeast extract. Table S5. Relative abundance of proteins belonging to Euryarchaeota. [file MBT2-11-1137-s001.pdf]

Desulfitobacterium contributes to the microbial transformation of 2,4,5-T by methanogenic enrichment cultures from a Vietnamese active landfill

Supporting information

Ute Lechner[1*], Dominique Türkowsky[2]*, Dinh Thi Thu Hang[3]*, Hassan Al-Fathi[1], Stefan Schwoch[1], Stefan Franke[1], Michelle-Sophie Gerlach[1], Mandy Koch[4], Martin von Bergen[2], Nico Jehmlich[2], Dang Thi Cam Ha[3]

[1]Martin-Luther University Halle-Wittenberg, Institute of Biology/Microbiology, [2]Helmholtz Centre for Environmental Research – UFZ, Leipzig, Department of Molecular Systems Biology, [3]Vietnamese Academy of Science and Technology, Hanoi, Vietnam; [4]Martin-Luther University Halle-Wittenberg, Institute of Chemistry/Food and Environmental Chemistry

*Authors contributed equally to this work.

This Supporting Information contains a description of the bioactive landfill in Bien Hoa, Vietnam, a short summary of the results of the primary and secondary enrichment cultures (Dinh et al. 2015) and Tables S1, S3, S4 and Figures S1 to S9.

Description of the bioactive landfill in Bien Hoa, establishment of enrichment cultures and results of 2,4,5-T degradation in the primary and secondary enrichment cultures

The bioactive landfill in Bien Hoa was situated 700 meters from the Dong Nai river on the West (Dang and Nguyen, 2012). The landfill consisted of four cells (BH1-BH4) containing 3,384 m^3 of herbicide and dioxin contaminated soil. The soil contaminated with polychlorinated dibenzo-*p*-dioxins and herbicides was well mixed with different agents such as rice straw, hulls, biosurfactants, nutrients, electron acceptors, electron donors and vitamins and was periodically supplied with water (moisture range from 16-24%). The 2 m thick soil layer was sealed above and below with four layers of bentonite and geological protection material and 50 cm of non-contaminated soil as the top layer, which was planted with grass. Six pipes per cell allowed gas evaporation. Samples were taken from cell 1 (BH1.1:10°57’783’’N, 106°49’388’’E) and from two positions of cell 4 (BH4.3: 10°57’774’’N, 106°49’387’’E; BH4.4:10°57’780’’N, 106°49’387’’E) at a depth of 1.5-1.7 m 36 months after the establishment of the landfill.

Collected samples were used for the preparation of primary microcosms (Dinh et al., 2015).They were prepared from soil (20 % w/v) of the sampling sites BH1.1, BH4.3 and BH4.4 in 40 ml of anoxic medium and incubated with 100 µM 2,4,5-T and a mixture of 5 mM pyruvate and 5 mM lactate as described in the Experimental Procedures.

2,4,5-T was completely removed within 2 weeks in all three enrichment cultures. The kinetics and pathways of transformation were studied in duplicate subcultures. 2,4,5-T disappeared within 10 days in all cultures. The subcultures BH1.1 and BH4.4 formed 2,4,5-TCP and 3,4-DCP as the main products and only low amounts of 3-chlorophenol and 2,5-dichlorophenol, whereas the subculture BH4.3 produced 2,5-dichlorophenol and 3-chlorophenol as the main products and only traces of 2,4,5-TCP and 3,4-DCP. This suggested that in the BH4.3 culture 2,4,5-T was first dechlorinated to 2,5-dichlorophenoxyacetic acid followed by the cleavage of the ether bond, which resulted in the formation of 2,5-dichlorophenol and finally 3-chlorophenol. In contrast, in subcultures BH1.1 and BH4.4 the ether bond of 2,4,5-T was initially cleaved followed by the reductive dechlorination of the intermediate 2,4,5-TCP. The promising transformation pathway of BH4.3 leading from 2,4,5-T to 3-chlorophenol changed during the further sub-cultivation to the transformation sequence described in the main text, strongly suggesting that a specific 2,4,5-T-dechlorinating organism was lost under the chosen cultivation conditions.

Table S1. Description of the 2,4,5-T enrichment culture transfers used for the experiments[a]

Enrichment culture(s)	Transfer number	Electron donor(s)	Reducing agent(s)	Acetate (2.5 mM)	Yeast extract (50 mg L^{-1})	Subject studied (data shown in)
BH1.1, BH4.3, BH4.4	1[b]	5 mM pyruvate + 5 mM lactate	0.4 mM Ti (III) citrate + FeS	+	+	Primary enrichment culture (Dinh et al., 2015)
BH1.1, BH4.3, BH4.4	2	5 mM pyruvate + 5 mM lactate	0.4 mM Ti (III) citrate + FeS	+	+	Dinh et al. (2015)
BH1.1, BH4.3, BH4.4	6	2.5 mM pyruvate or 2.5 mM lactate	0.4 mM Ti (III) citrate + FeS	+	+	Analysis of transformation products (Fig. 1, Table S1[c])
BH1.1, BH4.3, BH4.4	8	2.5 mM pyruvate or 2.5 mM lactate	0.4 mM Ti (III) citrate + FeS	+	+	PCR with *Desulfitobacterium*-specific primers (Fig. S2[c])
BH4.4	9	5 mM pyruvate	0.4 mM Ti (III) citrate + FeS	+	+	2,4,5-T- vs. 2,4,5-TCP transformation (Fig. S1[c])
BH1.1	11	5 mM pyruvate	0.4 mM Ti (III) citrate + FeS	-	+	Quantification of *Desulfitobacterium* 16S rRNA genes (Fig. 2)
BH1.1	11	5 mM pyruvate	0.4 mM Ti (III) citrate + FeS	-	-/+	Sequencing of 16S rRNA gene amplicons (Fig. S5[c])
BH1.1	11	5 mM pyruvate	0.4 mM Ti (III) citrate + FeS	-	-/+	Metaproteome analysis of cultures with and without 2,4,5-T and with and without yeast extract (Tables 2, 3, Fig. 6, Tables S2[c], S3[c], S4[c], Fig. S6[c], S7[c], S8[c])
BH1.1	11	5 mM pyruvate	0.4 mM Ti (III) citrate + FeS	-	-	Metaproteome analysis at different time points (Table S5[c], Fig. S9[c])
BH1.1	12	5 mM pyruvate	0.4 mM Ti (III) citrate + FeS	-	+	Substrate spectrum (Table 1)
BH1.1	12	5 mM pyruvate	1 mM Ti (III) citrate	-	+	Influence of bromoethane sulfonate on 2,4,5-T and 2,4,5-TCP transformation (Fig. 4)
BH1.1	15	5 mM pyruvate	1 mM Ti (III) citrate	-	-/+	Stoichiometry of pyruvate fermentation (Fig. 3, Fig. S3[c])
BH1.1	15	hydrogen	1 mM Ti (III) nitrilotriacetate	-	-	Hydrogen as electron donor for 2,4,5-T transformation (Fig. 5)
BH1.1	17	hydrogen	1 mM Ti (III) nitrilotriacetate	-	-	Hydrogen as electron donor for 2,4,5-TCP dechlorination

[a]Cultures were inoculated by 10 % (vol/vol) with the preceding transfer and supplemented with 50-200 µM 2,4,5-T; [b]primary microcosm; [c]in this Supporting Information

Table S2. Chlorinated phenoxyacetic acids and phenols (µM) identified and quantified by mass spectrometry after 10 days of cultivation in the 6th subculture of mixed cultures BH1.1, BH 3.4 and BH4.4 and abiotic controls incubated with 100-200 µM 2,4,5-T and either 2.5 mM pyruvate or 2.5 mM lactate. The main products, which exceeded the trace concentrations in the controls, are indicated in bold. n.d., not detected.

Mixed culture/ Co-substrate	BH1.1/ Lactate	BH1.1/ Pyruvate	BH4.3/ Pyruvate	BH4.4/ Lactate	BH4.4/ Pyruvate	Control/ Lactate	Control/ Pyruvate
Phenol	0.50	0.73	0.53	0.57	0.38	0.26	0.28
4-Chlorophenol	0.02	**2.09**	**1.35**	0.06	n.d.	0.04	0.04
2,5-Dichlorophenol	0.03	n.d.	n.d.	0.23	n.d.	n.d.	n.d.
2,4-Dichlorophenol	n.d.	n.d.	n.d.	0.10	n.d.	n.d.	n.d.
2,4,5-Trichlorophenol	**34.77**	**2.98**	**8.88**	**59.07**	**3.62**	0.41	1.01
3,4-Dichlorophenol	**0.47**	**32.94**	**1.78**	n.d.	**1.17**	n.d.	n.d.
2-/3-Chlorophenoxy acetic acid	0.11	n.d.	0.05	n.d.	n.d.	n.d.	n.d.
2,4-D	0.10	n.d.	0.02	0.11	n.d.	0.12	0.10
2,3-/3,4-D	0.20	n.d.	n.d.	0.24	n.d.	n.d.	0.17
2,4,5-T	82.26	182.65	53.08	190.82	59.05	106.12	115,32

Table S4. Protein groups upregulated by 2,4,5-T (adj. p<0.05) or unique in the presence of 2,4,5-T (with an abundance >median, p<0.05) with and/or without yeast extract.

Accession no. of meta-protein	Description of lowest common ancestor	Taxon of lowest common ancestor	log 2-fold change	
			Without YE[a]	With YE[a]
			+ 2,4,5-T vs. - 2,4,5-T	+ 2,4,5-T vs. - 2,4,5-T
**B8FVX4**	**Reductive dehalogenase**	***Peptococcaceae***	**Unique for 245T[c]**	**4.2**
**Q9ANS1**	**Reductive dehalogenase**	***Desulfitobacterium***	**Unique for 245T[d]**	**Unique for 245T[d]**
**518058910**	**ABC-type oligopeptide transport system, periplasmic component**	***Sedimentibacter* sp. B4**	**Unique for 245T[d]**	**Unique for 245T[e]**
**A0A098AWR7**	**Alcohol dehydrogenase, class IV**	***Clostridiales***	**3.0**	**3.1**
**A0A098B7J4**	**Uncharacterized protein conserved in bacteria**	***Desulfitobacterium hafniense***	**1.9**	**2.7**
**A0A098AW87**	**Formylmethanofuran dehydrogenase subunit E**	***Clostridiales***	**2.5**	**2.7[f]**
**G9XMD7**	**Electron transfer flavoprotein, beta subunit**	***Clostridiales***	**1.5[f]**	**1.9**
**A0A098AV40**	**ABC-type Fe3+-hydroxamate transport system, periplasmic component**	***Desulfitobacterium***	**2.7[f]**	**2.5**
**A4J9D6**	**Dissimilatory sulfite reductase (desulfoviridin), alpha and beta subunits**	**heterogeneous**	**2.7[f]**	**1.6**
**Q24S74**	**Putative cell wall-binding domain**	***Desulfitobacterium***	**1.3[f]**	**1.1**
**B8FVX9**	**Uroporphyrinogen-III methylase**	**heterogeneous**	**1.4[f]**	**1.9**
**A0A098AWK9**	**ABC-type dipeptide transport system, periplasmic component**	***Firmicutes***	**1.7[f]**	**1.6**

A0A098AWD4	Rubrerythrin	*Desulfitobacterium*	3.6	non-differential[g]
A0A098B095	Uncharacterized protein conserved in bacteria	*Peptococcaceae*	2.6	non-differential[g]
A0A098AYA1	Ferritin-like protein	heterogeneous	2.2	n.d.
A0A098AVC4	Peroxiredoxin	heterogeneous	2	n.d.
518059289	UDP-N-acetylglucosamine:LPS N-acetylglucosamine transferase	*Sedimentibacter sp. B4*	1.6	non-differential
Q24S05	ABC-type molybdate transport system, periplasmic component	*Desulfitobacterium*	1.5	non-differential[g]
518059037	hypothetical protein	*Sedimentibacter* sp. B4	1.3	non-differential
B8J261	Aerobic-type carbon monoxide dehydrogenase, large subunit CoxL/CutL homologs	*Desulfovibrionales*	1.3	non-differential
D7GSJ3	Uncharacterized protein	butyrate-producing bacterium SS3/4	1.1	n.d.
A0A075KDX4	Diaminopimelate epimerase	*Pelosinus*	1.0	non-differential
A0A098B093	Electron transfer flavoprotein, beta subunit	*Desulfitobacterium*	non-differential	4
A0A0W1JIJ6	Electron transfer flavoprotein, beta subunit	*Desulfitobacterium hafniense*	non-differential	4
A4J6N7	ABC-type uncharacterized transport system, periplasmic component	*Desulfotomaculum reducens*	n.d.	2.9
G7WGR8	Enoyl-CoA hydratase/carnithine racemase	*Desulfosporosinus orientis*	non-differential	2.5

A4J179	Ketol-acid reductoisomerase	heterogeneous	n.d.	2.4
A4J0T5	Predicted NADH:ubiquinone oxidoreductase, subunit RnfC	*Desulfotomaculum*	n.d.	2.3
A0A099RZ05	Aspartokinases	*Clostridiales*	n.d.	2.3
A4J491	Tryptophan synthase beta chain	heterogeneous	n.d.	2.3
A0A099S4D2	Tfp pilus assembly protein PilF	*Clostridium* sp. HMP27	n.d.	2.1
A3DJF7	Imidazoleglycerol-phosphate synthase	*Clostridia*	n.d.	2.1
C6BRY3	Lysine 2,3-aminomutase	heterogeneous	n.d.	2.1
Q9AF51	Dissimilatory sulfite reductase (desulfoviridin), alpha and beta subunits	*Desulfotomaculum*	n.d.	2
I8RKQ8	Glutamate mutase epsilon subunit	*Firmicutes*	non-differential	2
518057529	Glutamine synthetase	*Sedimentibacter* sp. B4	non-differential	2
F6B3Q5	MET3, ATP sulfurylase	heterogeneous	non-differential	1.9
F6DSR0	6Fe-6S prismane cluster-containing protein	heterogeneous	non-differential	1.9
A4J5F1	Electron transfer flavoprotein, alpha subunit	*Clostridiales*	-1.5	1.9
F5CPC6	Dissimilatory sulfite reductase (desulfoviridin), alpha and beta subunits	*Desulfotomaculum*	n.d.	1.9
K8EIH2	Dissimilatory sulfite reductase (desulfoviridin), alpha and beta subunits	*Desulfotomaculum*	non-differential[g]	1.9
A4J9D5	Dissimilatory sulfite reductase (desulfoviridin), alpha and beta subunits	*Desulfotomaculum*	non-differential[g]	1.8
Q6V113	Dissimilatory sulfite reductase (desulfoviridin), alpha and beta	heterogeneous	non-differential	1.8

	subunits			
L1QKX1	Enoyl-CoA hydratase/carnithine racemase	*Clostridiales*	n.d.	1.7
B0K3B8	Propanediol dehydratase, small subunit	heterogeneous	non-differential[g]	1.7
A0A099RRJ4	Uncharacterized proteins involved in stress response, homologs of TerZ and putative cAMP-binding protein CABP1	*Clostridiales*	n.d.	1.7
C2MDL6	Uncharacterized flavoproteins	*Porphyromonas*	n.d.	1.7
A6YD02	Ferredoxin-related protein.	*Peptococcaceae*	non-differential	1.7
A4J273	Indolepyruvate ferredoxin oxidoreductase, alpha and beta subunits	*Peptococcaceae*	non-differential	1.7
F6B613	3-Polyprenyl-4-hydroxybenzoate decarboxylase	*Clostridiales*	n.d.	1.6
A0A0F2PK70	Electron transfer flavoprotein, alpha subunit	*Clostridiales*	non-differential	1.6
Q8VRU2	Succinate dehydrogenase/fumarate reductase, flavoprotein subunit	heterogeneous	non-differential	1.6
F6B3Q3	Succinate dehydrogenase/fumarate reductase, flavoprotein subunit	heterogeneous	non-differential	1.6
K6TUI1	Coenzyme F420-reducing hydrogenase, gamma subunit	*Methanobacterium*	non-differential	1.5
A0A127EIS5	Cysteine synthase	*Clostridium perfringens*	n.d.	1.5
K8EAK4	2-polyprenylphenol hydroxylase and related flavodoxin oxidoreductases	*Peptococcaceae*	n.d.	1.5
A4J274	Succinate dehydrogenase/fumarate reductase, flavoprotein subunit	heterogeneous	non-differential	1.4
A0A099RLP7	-	heterogeneous	n.d.	1.4
A0A069A552	Uncharacterized conserved protein related to	*Clostridioides difficile*	n.d.	1.2

	dihydrodipicolinate reductase			
A0A0B3VUX3	Na+-dependent transporters of the SNF family	*Terrisporobacter othiniensis*	n.d.	1.2
A0A099SB78	Metal-dependent amidase/aminoacylase/carboxypeptidase	*Clostridium*	n.d.	1.1
A0A099S4B8	Phenylalanyl-tRNA synthetase beta subunit	heterogeneous	n.d.	1.0
A0A010Z2D4	ABC-type sugar transport system, periplasmic component	*Clostridiales*	Unique for 245T	n.d.
A0A098B703	GMP synthase, PP-ATPase domain/subunit	heterogeneous	Unique for 245T	non-differential
A8MJQ2	ABC-type branched-chain amino acid transport systems, periplasmic component	*Clostridiales*	Ident	Unique for 245T
A0A098B022	Methionine synthase I, cobalamin-binding domain	*Clostridiales*	non-differential	Unique for 245T
A0A0L6W010	Pyruvate:ferredoxin oxidoreductase and related 2-oxoacid:ferredoxin oxidoreductases, gamma subunit	*Clostridiales*	non-differential	Unique for 245T
A4J0J6	Pyruvate:ferredoxin oxidoreductase and related 2-oxoacid:ferredoxin oxidoreductases, gamma subunit	*Clostridiales*	n.d.	Unique for 245T
A4J6Q0	ABC-type branched-chain amino acid transport systems, periplasmic component	*Desulfotomaculum reducens*	n.d.	Unique for 245T

2,4,5-T, 2,4,5-trichlorophenoxyacetic acid; adj. p, Benjamini-Hochberg adjusted p-value from student's t-test; n.d., protein not detectable in both conditions; non-differential, protein with a fold change that was not significant; Ident, protein was only identified but not quantifiable in at least one condition; [a]yeast extract; [b]data in bold indicate upregulation by 2,4,5-T in both conditions, in the absence and presence of yeast extract; [c]protein abundance >median of all proteins in this condition; [d]protein abundance <median of all proteins in this condition; [e]protein abundance <median-1SD (standard deviation) of all proteins in this condition; [f]adjusted p-value >0.05, raw p-value <0.05; [g]unique for 245T, but p>0.05, therefore regarded as non-differential.

Table S5. Relative abundance of protein groups belonging to *Euryarchaeota*. Enrichment cultures were analysed following sub-cultivation with and without 2,4,5-T, both in the presence and absence of yeast extract (YE).

Accession no. of meta-protein	Description of lowest common ancestor	log2 average abundance[a]			
		Without YE[a]		With YE	
		+ 2,4,5-T	- 2,4,5-T	+ 2,4,5-T	- 2,4,5-T
A0A090I5T3	Methyl-coenzyme M reductase II gamma subunit MrtG	3.2	3.4	4.6	Ident
M1Q5Y4	UPF0210 protein MmTuc01_0091	8.2	8.5	6.5	Ident
A0A089Z7S3	F420-dependent methylenetetrahydromethanopterin dehydrogenase	Ident	5.3	6.3	5.5
A0A090I2T7	Methyl-coenzyme M reductase I subunit gamma	Ident	4.9	5.1	5.8
A0A090I2G6	Methyl-coenzyme M reductase alpha subunit McrA	n.d.	4.0	5.2	4.8
A0A090I573	5,10-methylenetetrahydromethanopterin reductase	4.9	5.0	6.4	6.1
A0A089ZFI9	F420-non-reducing hydrogenase subunit A	n.d.	4.6	4.2	3.9
K6TUI1	Coenzyme F420-reducing hydrogenase, gamma subunit (Fragment)	n.d.	Ident	4.1	2.6
P60238	F420-non-reducing hydrogenase iron-sulfur subunit D	Ident	Ident	1.9	n.d.
A0A090I5H7	Tetrahydromethanopterin S-methyltransferase subunit H	4.4	4.8	5.0	4.9
A0A090I2G3	Tetrahydromethanopterin S-methyltransferase subunit A	n.d.	Ident	3.2	3.4
A0A090I7W3	Methyl-coenzyme M reductase I subunit beta	n.d.	4.7	5.6	5.6

U6EEC5	Methyl-coenzyme M reductase I subunit beta	n.d.	4.7	5.7	5.6
A0A089ZDH4	Elongation factor 1-alpha	n.d.	2.9	6.7	7.1
A0A090I305	Coenzyme F420 hydrogenase alpha subunit FrhA	n.d.	n.d.	5.0	Ident
A0A090I2T3	Methyl-coenzyme M reductase II beta subunit MrtB	4.1	4.3	3.8	4.0
O50374	FprB	n.d.	4.5	n.d.	n.d.
A0A089ZJ21	Methyl-coenzyme M reductase II alpha subunit MrtA	n.d.	n.d.	3.4	n.d.
A0A089ZUU9	CoB--CoM heterodisulfide reductase subunit C HdrC1	Ident	Ident	n.d.	3.2
A0A0E3NVN7	Ribose-5-phosphate isomerase A	9.1	9.7	7.4	8.0
A0A090I5C2	V-type ATP synthase beta chain	n.d.	n.d.	Ident	3.7
A0A090I3G7	CoB--CoM heterodisulfide reductase subunit A HdrA2	n.d.	Ident	Ident	3.4
K2R1R4	Peroxiredoxin	5.7	5.8	Ident	Ident
A0A090IAH6	Thermosome subunit	n.d.	Ident	n.d.	n.d.
K2R229	Catalase-peroxidase	7.5	n.d.	n.d.	n.d.
A0A089ZH29	Putative secreted protein	4.0	n.d.	n.d.	n.d.
A0A090I7T8	Peptidyl-prolyl cis-trans isomerase	2.9	Ident	n.d.	n.d.

YE, yeast extract; 2,4,5-T, 2,4,5-trichlorophenoxyacetic acid; n.d., protein not detectable; Ident, protein was only identified but not quantifiable; [a]log2-fold change of the median of each condition

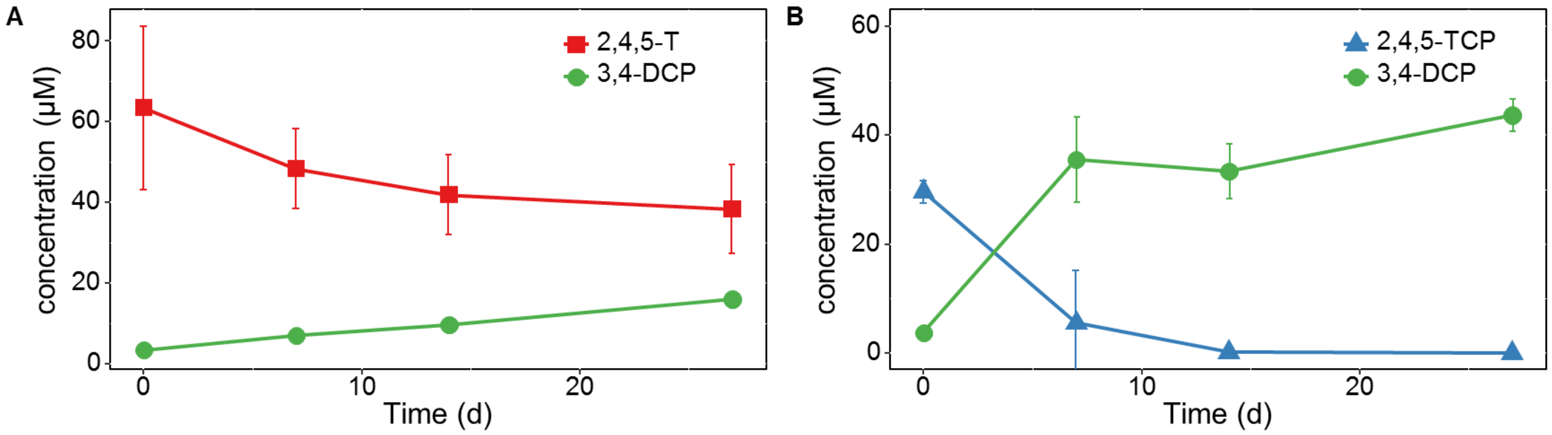

Fig. S1. Formation of 3,4-dichlorophenol (3,4-DCP) in enrichment culture BH4.4, supplemented with 2,4,5-T (A) or 2,4,5-trichlorophenol (2,4,5-TCP) (B) and 5 mM pyruvate (mean values of triplicate cultures and SD). Note that during the conversion of 2,4,5-T to 3,4-DCP the intermediate 2,4,5-TCP was detected in traces only (not shown).

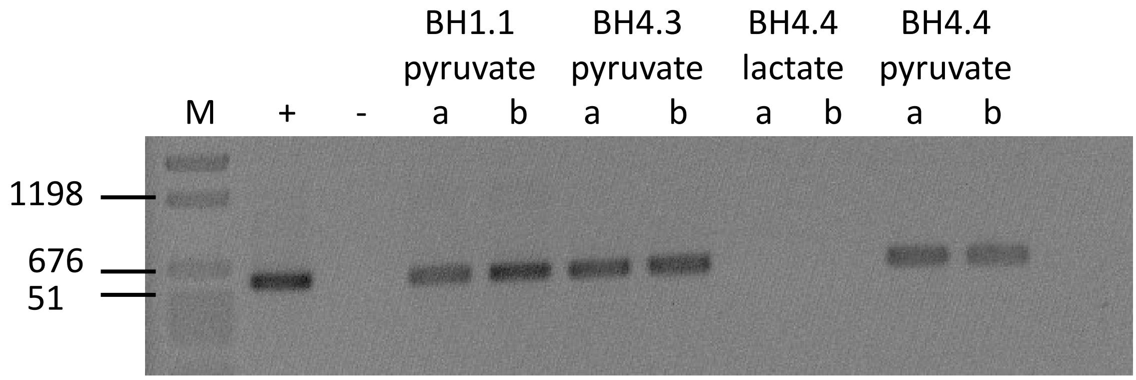

Fig. S2. Detection of *Desulfitobacterium* in the 8th subculture of mixed cultures BH1.1, BH4.3 and BH4.4 in duplicate cultures (a, b) incubated with 100 µM 2,4,5-T and pyruvate or lactate. DNA was extracted after 4 weeks of incubation, when degradation products (2,4,5-TCP and 3,4-DCP in pyruvate-fed cultures and only 2,4,5-TCP in lactate-fed cultures) were formed. PCR products were obtained from DNA extracts using the primer pair DES436f/DES1027r. M: pGEM marker (bp), +: genomic DNA of *Desulfitobacterium hafniense* TCP-A, -: no template. Sequencing of the PCR products revealed >99.5 % identity to *Desulfitobacterium hafniense*.

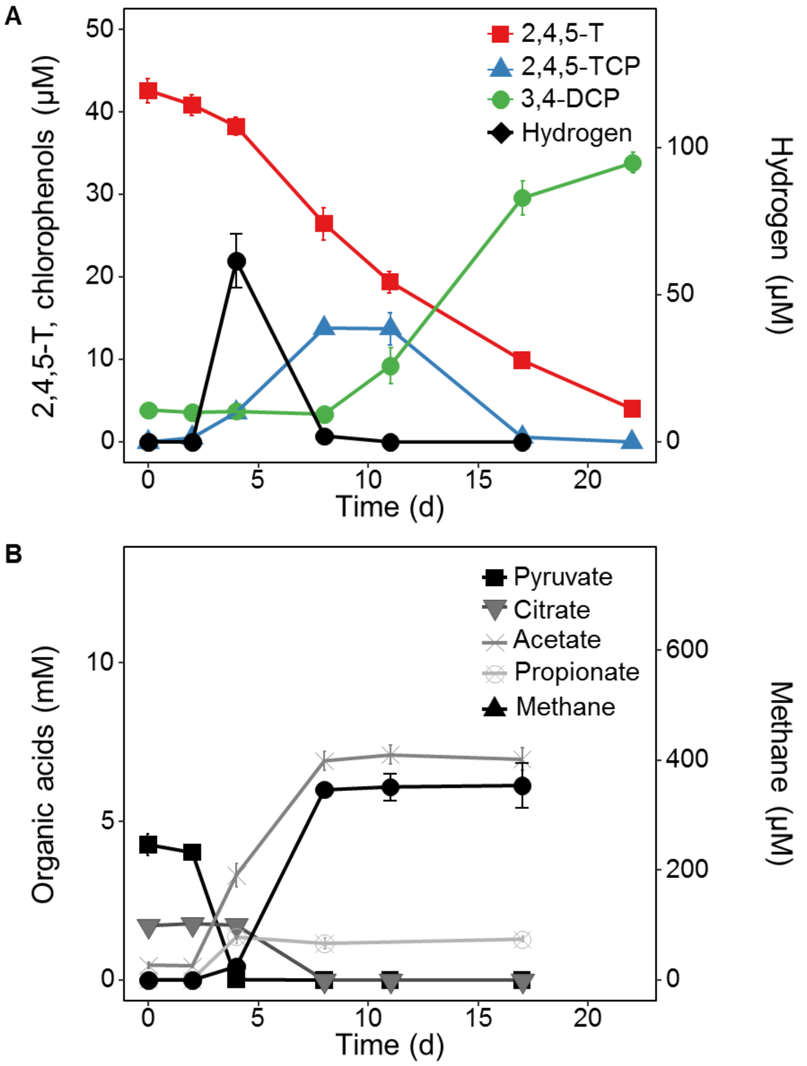

Fig. S3. Transformation of 2,4,5-T (A) and fermentation of pyruvate and citrate (B) in enrichment culture BH1.1 cultivated in the presence of yeast extract. Intermediate formation of hydrogen is shown for clarity in A whereas formation of other fermentation products and methane are given in B. Mean value and SD of triplicate cultures. Lactate (0.2 mM, possibly an impurity of pyruvate) was detected at the beginning and disappeared already after 4 days (data not shown).

a) $3\ CH_3\text{-}CO\text{-}COOH + H_2O \longrightarrow 2\ CH_3\text{-}COOH + CH_3\text{-}CH_2\text{-}COOH + 2\ CO_2$

3.6 mM* *2.4 mM* **1.2 mM**

b) $CH_3\text{-}CO\text{-}COOH + 2\ H_2O \longrightarrow CH_3\text{-}COOH + CO_2 + H_2$

0.6 mM* *0.6 mM* *0.6 mM*

c) $HOOC\text{-}CH_2\text{-}C(COOH)(OH)\text{-}CH_2\text{-}COOH + H_2O \longrightarrow 1.7\ CH_3\text{-}COOH + 0.3\ CH_3\text{-}CH_2\text{-}OH + 2\ CO_2 + 0.4\ H_2$

1.8 mM *3.1 mM* *0.7 mM*

d) $CO_2 + 4\ H_2 \longrightarrow CH_4 + 2\ H_2O$

1. 2 mM **0.31 mM**

Fig. S4. Fermentation of pyruvate (a, b) and citrate (c) and methanogenesis (d) by the enrichment culture. The mass balance was calculated on the basis of the determined concentrations of pyruvate and citrate at day 0 and of the products propionate and methane at day 8 (Fig. 3B) (given in bold below the equations). The concentration of acetate and hydrogen is indicated in italics and was calculated according to the reaction stoichiometry. Ethanol and CO_2 were not analysed. a) Pyruvate is fermented via the propionic acid fermentation pathway. Two moles of pyruvate are oxidized to two moles each of CO_2 and acetyl-CoA, the latter of which can be converted to two moles of acetate in an energy-conserving reaction. The formed NADH can be re-oxidized via the formation of propionic acid from a third mole of pyruvate. The detected 1.2 mM propionate suggests that 3.6 mM of the total added pyruvate (4.2 mM) were converted by this fermentation stoichiometry. b) Pyruvate (the remaining 0.6 mM) is proposed to be oxidized to acetyl-CoA by pyruvate:ferredoxin oxidoreductase or pyruvate formate lyase, thereby forming either reduced ferredoxin or formate, which both can deliver hydrogen by the action of hydrogenases or the formate hydrogen-lyase complex, respectively. c) Citrate fermentation via the citrate lyase reaction results in the formation of acetate, CO_2, ethanol and hydrogen (Walther et al., 1977). d) Methane formation by hydrogenotrophic methanogens.

The total concentration of acetate determined after 8 days (6.5 mM, Fig. 3) exceeded only slightly the theoretical sum (6.1 mM) obtained from the fermentation of pyruvate and citrate (equations a-c), supporting the proposed fermentation reactions. Additional processes such as acetogenesis or the oxidation of the proposed intermediate ethanol might have an additional limited influence on the acetate concentration. The presence of the CO dehydrogenase/acetyl CoA synthase as well of an alcohol dehydrogenase class IV in the metaproteome supports these assumptions (Table 2 and Supporting Information, Table S2), whereas the ether cleavage of the micromolar concentrations of 2,4,5-T might contribute only negligible amounts of acetate.

Hydrogen forming (equation b, c) and consuming processes (equation d) are well balanced with only a minimal excess of hydrogen formation (0.1 mM), which might be sufficient to drive 2,4,5-T ether cleavage and dechlorination. The low level of detected hydrogen in the enrichment culture also supports the proposed metabolic routes.

*indicates that portions of the initial 4.2 mM pyruvate were assigned to reactions a and b according to the determined concentration of propionate formed in process a.

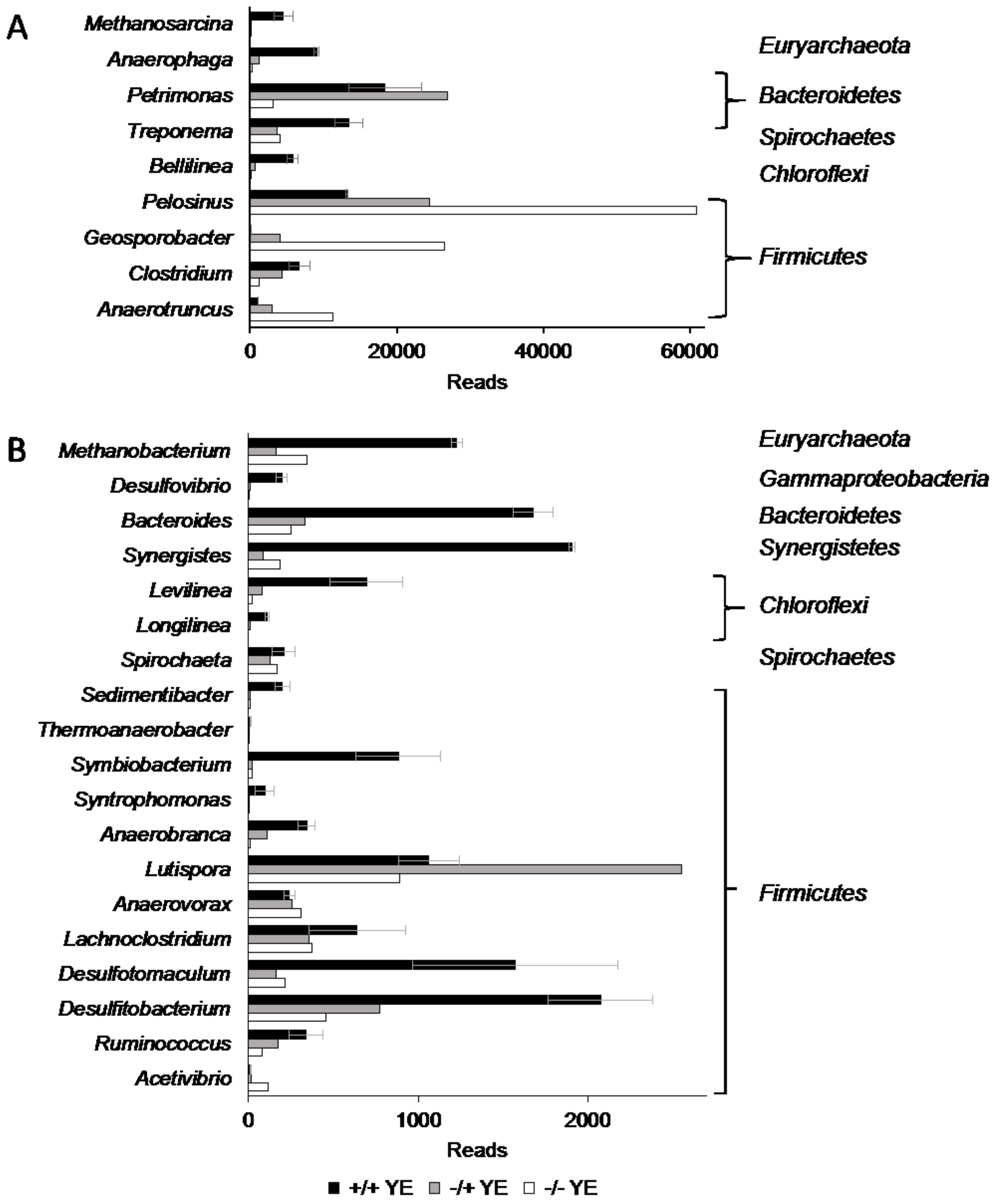

Fig. S5. Genus-level distribution based on amplicon sequencing of 16S rRNA genes. The read numbers of 16S rRNA gene amplicons are shown from BH1.1 cultures enriched with 2,4,5-T and pyruvate with three different histories of yeast extract addition. The cultures were grown in the presence of 50 mg l^{-1} yeast extract over 11 transfers (+/+YE, mean value and SD of two cultures) or 8 transfers with yeast extract, followed by two transfers without yeast extract and a third transfer with (-/+YE) or without yeast extract (-/-YE). Genera represented by more or less than 4000 reads in each sample are summarized in A and B, respectively. Genera, which were represented by less than 100 (< 0.25 % of total) reads in each sample were omitted.

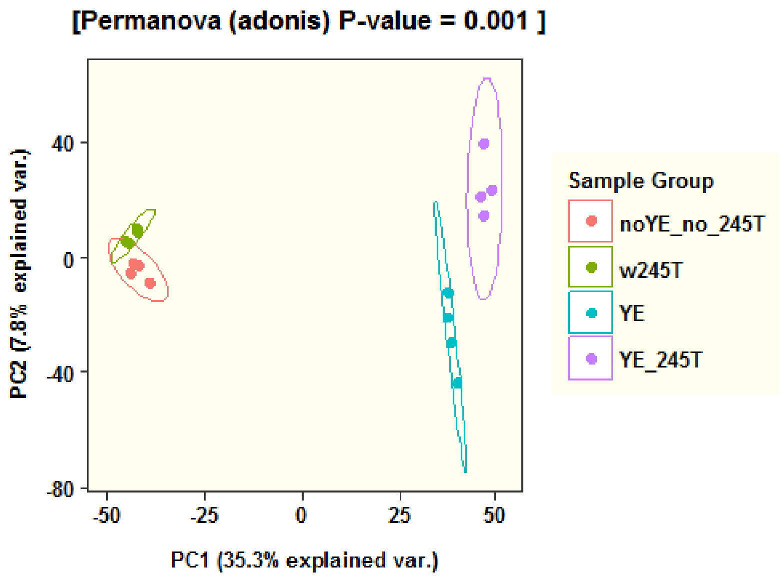

Fig. S6. PCA-Plot of all detected proteins of the metaproteomic analysis of four replicates and of four different conditions. noYE, without yeast extract; YE, with yeast extract; no_245T, without 2,4,5-T; 245T, with 2,4,5-T. Plots were generated with an in-house R-script using the packages gplots, ggplot2, ggbiplot, dplyr, miscTools and vegan.

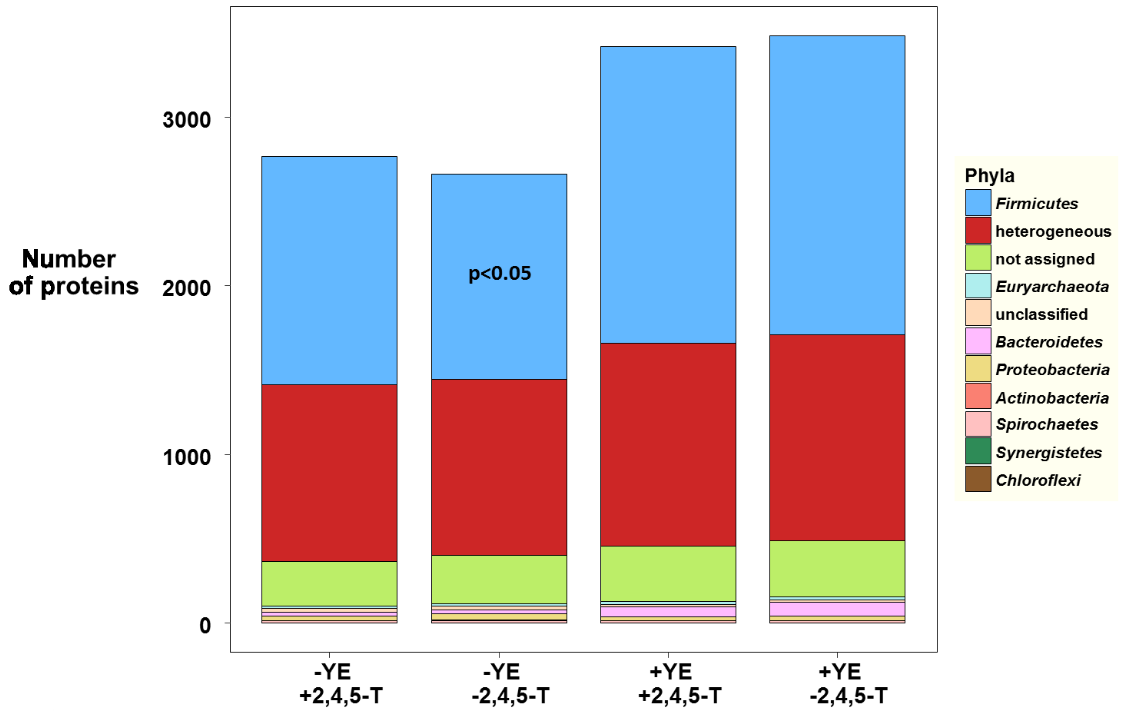

Fig. S7. Phylogenetic distribution of proteins. Abundances calculated from the number of proteins detected for each phylum in the mixed cultures using different cultivation conditions (four replicate cultures per condition). Cultures were supplemented with 2,4,5-trichloropenoxyacetic acid (+2,4,5-T) and without (-2,4,5-T). One set of cultures each was incubated with yeast extract (+YE) and without (- YE). P-value indicates significant difference between +2,4,5-T and -2,4,5-T. Plots were generated with an in-house R-script using the packages gplots, ggbiplot.

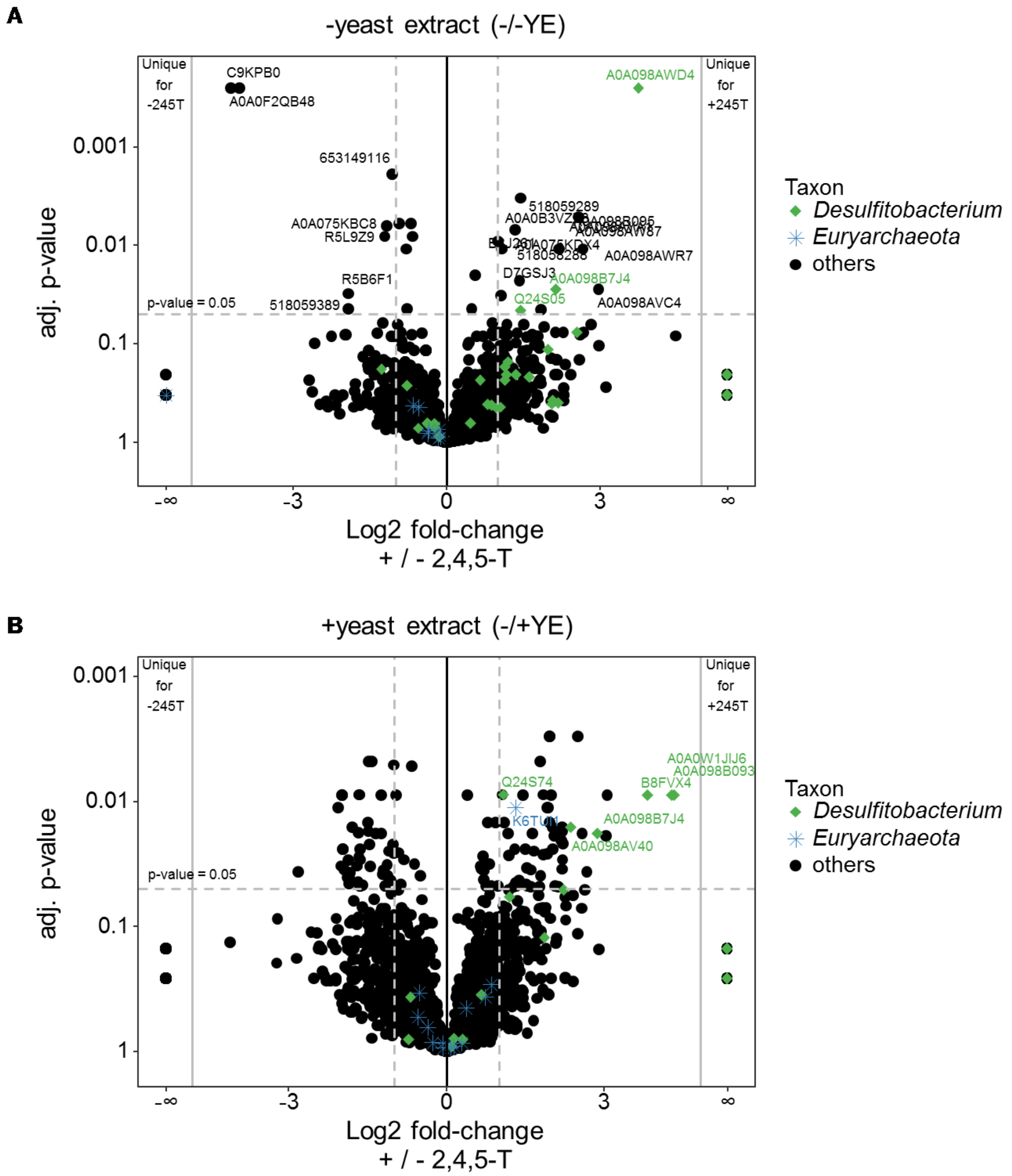

Fig. S8. Log2-fold change and Benjamini-Hochberg adjusted p-values of all quantifiable and unique meta-proteins compared for +2,4,5-T and -2,4,5-T, without YE (A) and with YE (B). The logarithmic ratios of protein amounts in the mixed culture BH1.1 were plotted against Benjamini-Hochberg-adjusted p-values of the t test performed from four replicates. The dotted lines indicate thresholds set for regulation (log2 fold change >1 and <-1 indicating a 2-fold higher abundance with and without 2,4,5-T) and the significance (adj. p-value <0.05). Significantly higher abundant proteins are labelled with the accession number of the meta-protein. Proteins with a fold change of ∞ or -∞ are unique to one of the conditions. A list of these proteins can be found in Table S3. Plots were generated with an in-house R-script using the packages gplots, ggplot2, ggbiplot, dplyr, miscTools and vegan.

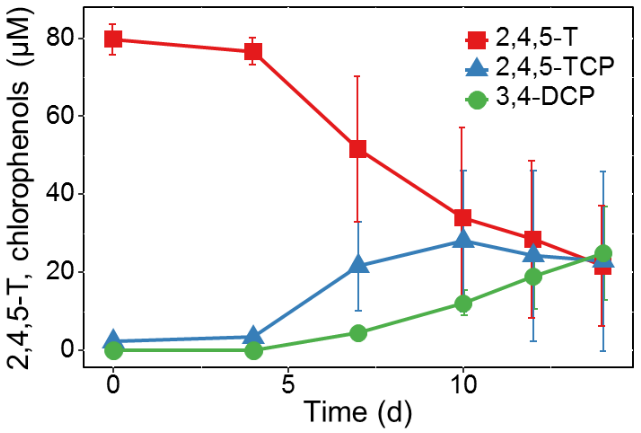

Fig. S9. Transformation of 2,4,5-T by mixed culture BH1.1 in the absence of yeast extract. The pre-culture also did not receive yeast extract (-/-YE). Samples for metaproteome analyses were taken after 7 and 14 days. Mean values and SD of triplicate cultures.

References:

Dang, T.C.H., and Nguyen, B.H. (2012) Treatment of dioxin contaminated soils by bioremediation. Patent no. 10246, Vietnam

Dinh, T.T.H., Dang, T.C.H., Dao, T.N.A., Phung, K.H.C., Pöritz, M., and Lechner, U. (2015) POP-dehalogenating microbes in an activated landfill treating herbicide- and dioxin-contaminated soil. *Organohalogen Compounds* **77**: 703-706.

Walther, R., Hippe, H., and Gottschalk, G. (1977) Citrate, a specific substrate for the isolation of *Clostridium sphenoides*. *Appl Environ Microbiol* **33**: 955-962.
